# Discovery of a Potent and Orally Active Dual GPBAR1/CysLT_1_R Modulator for the Treatment of Metabolic Fatty Liver Disease

**DOI:** 10.3389/fphar.2022.858137

**Published:** 2022-04-25

**Authors:** Stefano Fiorucci, Pasquale Rapacciuolo, Bianca Fiorillo, Rosalinda Roselli, Silvia Marchianò, Cristina Di Giorgio, Martina Bordoni, Rachele Bellini, Chiara Cassiano, Paolo Conflitti, Bruno Catalanotti, Vittorio Limongelli, Valentina Sepe, Michele Biagioli, Angela Zampella

**Affiliations:** ^1^ Department of Medicine and Surgery, University of Perugia, Perugia, Italy; ^2^ Department of Pharmacy, University of Naples “Federico II”, Naples, Italy; ^3^ Faculty of Biomedical Sciences, Euler Institute, Università della Svizzera italiana (USI), Lugano, Switzerland

**Keywords:** cysteinyl-leukotriene-receptor 1, g-protein coupled bile acid receptor 1, nonalcoholic fatty liver disease, nonalcoholic steatohepatitis (NASH), liver inflammation, REV5901 derivatives

## Abstract

Nonalcoholic fatty liver disease (NAFLD) and nonalcoholic steatohepatitis (NASH) are two highly prevalent human diseases caused by excessive fat deposition in the liver. Although multiple approaches have been suggested, NAFLD/NASH remains an unmet clinical need. Here, we report the discovery of a novel class of hybrid molecules designed to function as cysteinyl leukotriene receptor 1 (CysLT_1_R) antagonists and G protein bile acid receptor 1 (GPBAR1/TGR5) agonists for the treatment of NAFLD/NASH. The most potent of these compounds generated by harnessing the scaffold of the previously described CystLT_1_R antagonists showed efficacy in reversing liver histopathology features in a preclinical model of NASH, reshaping the liver transcriptome and the lipid and energy metabolism in the liver and adipose tissues. In summary, the present study described a novel orally active dual CysLT_1_R antagonist/GPBAR1 agonist that effectively protects against the development of NAFLD/NASH, showing promise for further development.

## 1 Introduction

Nonalcoholic fatty liver disease (NAFLD) is a highly prevalent human disorder affecting approximately one quarter of the population worldwide. NAFLD is characterized by an excessive fat deposition in the liver and is often considered the liver manifestation of a metabolic syndrome caused by chronic exposure to a high caloric intake and a sedentary lifestyle. Since insulin resistance is found as the main causative factor, NAFLD prevalence increases dramatically among patients with type 2 diabetes (T2DM), obesity, and dyslipidemia (Younossi et al., 2016; Ginès et al., 2021). NAFLD is categorized into simple steatosis or nonalcoholic fatty liver (NAFL) and nonalcoholic steatohepatitis (NASH). Although NAFL is generally considered a benign, nonprogressive liver disease, NASH is histologically typified by the presence of steatohepatitis, hepatocytes ballooning, and fibrosis carrying on significant risk for developing liver cirrhosis and hepatocellular carcinoma (Estes et al., 2018a; Estes et al., 2018b; Fiorucci and Distrutti 2022). In addition to an increased risk of developing liver cirrhosis, a large proportion of NAFLD/NASH patients are at risk of developing an atherosclerotic lipid profile and an atherosclerotic cardiovascular disease (CVD) (Lonardo et al., 2018; Sinn et al., 2020) and are at increased risk of developing fatal and nonfatal ischemic complications of CVD (Targher et al., 2016; Fiorucci and Distrutti 2022). Despite that the two components of the disease evolve sometimes independently, a correlation exists among the severity of hepatic fibrosis, a measure of NASH, and liver- and CVD-related mortality. It is increasingly recognized that NAFLD/NASH is associated with a state of liver inflammation. In the liver, the influx of inflammatory mediators from the intestinal microbiota and damaged/necrotic hepatocytes activates the Kupffer cells, the liver-resident macrophages, that, in turn, promote an inflammatory and fibrogenic response by inducing the trans-differentiation of quiescent hepatic stellate cells (HSC) into activated myofibroblasts, which are ultimately responsible for excessive extracellular matrix deposition (Trivedi et al., 2021). Despite that fibrosis is the main determinant of patients’ outcomes (Lee and Friedman 2022), the hepatic inflammatory response is one of the major drivers of disease progression from NAFLD to NASH (Biagioli and Fiorucci 2021), and specific anti-inflammatory approaches have been developed to treat inflammation and inflammation-driven fibrosis as either a mono-therapy or a combination therapy (Huby and Gautier 2021).

Several mediators are involved in the development of liver inflammation and fibrosis in NAFLD/NASH patients. Among the several targets, the cysteinyl leukotriene receptor 1 (CysLT_1_R) and the bile acid receptor GPBAR1 have been shown to be involved in disease development in several animal models of NAFLD/NASH. Of relevance, CysLT_1_R and GPBAR1 are expressed by the liver resident macrophages, the Kupffer cells (Keitel et al., 2007; Keitel et al., 2008; Fiorucci and Distrutti 2019; Fiorucci et al., 2021), and the HSC (Huber et al., 1989; Uemura et al., 1994; Clària et al., 1998; Horrillo et al., 2007; El-Swefy and Hassanen 2009; Kurtoğlu et al., 2019; Pu et al., 2021). Activation of these receptors in Kupffer cells promotes opposite effects. Thus, although CysLT_1_R promotes an inflammatory response, GPBAR1 agonists attenuate the production of inflammatory mediators through various molecular mechanisms, suggesting that CysLT_1_R antagonists might synergize with GPBAR1 agonists (Biagioli et al., 2017; Biagioli et al., 2019; Shi et al., 2020) in reducing liver inflammation. Additionally, the two receptors are expressed by several metabolically active tissues, including the white adipose tissues (WAT) exerting antagonistic effects on adipocytes. Thus, although CysLT_1_R agonism promotes insulin resistance and WAT inflammation, GPBAR1 exerts anti-inflammatory activities and promotes the browning of adipocytes and expression of anti-obesogenic targets such as UCP1, PGC1α, and PPARα (Carino et al., 2017b; Carino et al., 2017a; Carino et al., 2019b). Based on this background, we hypothesized that the development of dual CysLT_1_R antagonists/GPBAR1 agonists will be beneficial in treating NAFLD/NASH.

Following this general concept, we have recently envisaged in REV5901, a well-characterized CysLT_1_R antagonist, a privileged chemical scaffold for the development of dual CysLT_1_R antagonists/GPBAR1 agonists (Biagioli et al., 2020b), reporting the first family of derivatives improved in their synthetic accessibility and in their pharmacokinetic profiles (Fiorillo et al., 2021).

Indeed, extensive computations on CysLT_1_R and GPBAR1 structure assisted by *in-vitro* assays allowed the identification of the molecular bases for the simultaneous modulation of both receptors, setting up the springboard toward the identification of suitable drug candidates.

**FIGURE 1:**
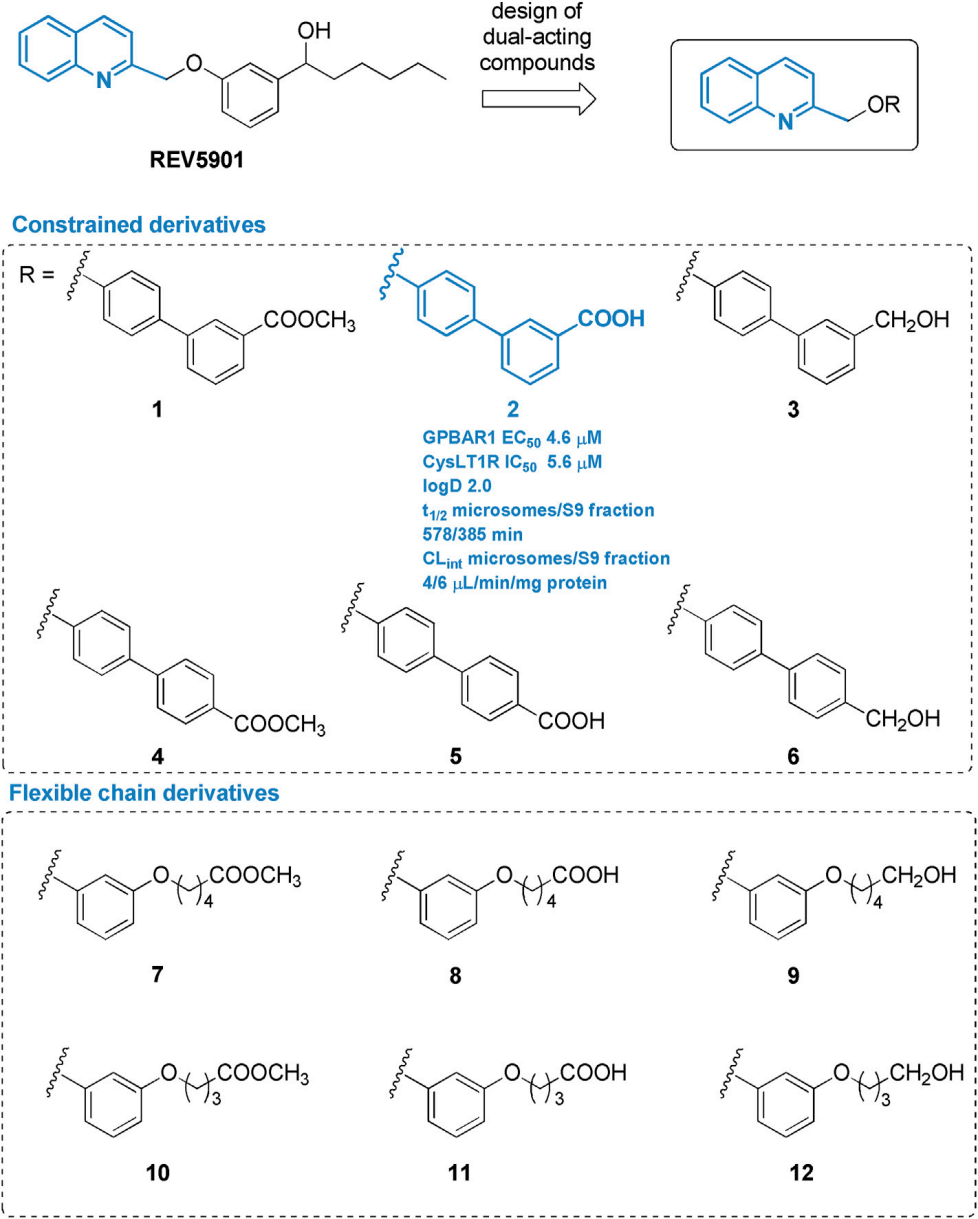
Chemical structure of derivatives identified in this study.

Pursuing this strategy and exploring further modifications on REV5901s quinoline scaffold (Figure 1), two sets of derivatives have been designed and synthetized by introducing a linker (rigid-biphenyl ring and flexible-alkyl chain) between the quinoline moiety and a polar or negatively charged end-group (Figure 1), able to intercept, through a water molecule-mediated H-bond, Arg79^2.60^ (superscripts refer to Ballesteros-Weinstein numbering) (Ballesteros and Weinstein 1995) in CysLT_1_R (Fiorillo et al., 2021), identifying several selective CysLT_1_R antagonists (compounds 7–12) and compound 2, a potent dual CysLT_1_R antagonist/GPBAR1 agonist, endowed with an excellent pharmacodynamic and pharmacokinetic profile. Assisted by a deep *in vivo* pharmacological evaluation, here we present, in a proof-of-concept study, the first evidence that dual modulation of CysLT_1_R/GPBAR1 represents a new armamentarium in NASH pharmacological treatment. The new chemical entity, compound 2, effectively reverses liver inflammation and fibrosis in a validated model of NASH and represents the first in class of a novel series of CysLT_1_R antagonists/GPBAR1 agonists.

## 2 Materials and Methods

### 2.1 General Experimental Methods

High-resolution ESI-MS spectra were performed with a LTQ-orbitrap spectrometer (Thermo Fisher Scientific, Waltham, MA, United States). NMR spectra were obtained on Bruker 400 spectrometer (^1^H at 400, ^13^C at 100 MHz) equipped with a Sun hardware and recorded in CDCl_3_ (*δ*_H_ = 7.26 and *δ*_C_ = 77.0 ppm) and CD_3_OD (*δ*_H_ = 3.30 and *δ*_C_ = 49.0 ppm). *J* are in hertz and chemical shifts (*δ*) are reported in ppm and referred to as CHCl_3_ and CHD_2_OD as internal standards. HPLC was performed using an Agilent 1260 Infinity II equipped with a Quaternary Pump VL model G7111A, a manual Rheodyne injector, and a Diode Array Detector WR model G7115A (Agilent Technologies, Santa Clara, California, United States). Reaction progress was monitored via thin-layer chromatography (TLC) on Alugram^®^ silica gel G/UV254 plates. Silica gel MN Kieselgel 60 (70–230 mesh) from Macherey-Nagel Company was used for column chromatography. All chemicals were obtained from Zentek S. r.l. (Milano, Italy) or Sigma Aldrich (St. Louis, Missouri, United States), and reagents were used as supplied from commercial sources with the following exceptions. Tetrahydrofuran was distilled from calcium hydride immediately prior to use. All reactions were carried out under argon atmosphere using flame-dried glassware. The purity of all the intermediates, checked by ^1^H NMR, was greater than 95%. The purity of tested compounds was determined to be always greater than 95% by analytical HPLC analysis as reported for each compound.

### 2.2 Chemistry

Williamson reaction. The 2-(cloromethyl)quinoline was added to a stirred mixture of the phenol (1.0 eq), K_2_CO_3_ (2.5 eq), and dry DMF. The reaction mixture was stirred at 100°C for 12 h. The mixture was diluted with H_2_O and extracted with EtOAc (3 × 30 mL). The combined organics were washed with brine, dried (Na_2_SO_4_), concentrated, and purified by flash chromatography.

Basic hydrolysis. An aliquot of esters was dissolved in MeOH/H_2_O (1:1 v/v) and treated with NaOH (5 mol eq.) at 0°C. The resulting mixture was stirred under reflux for 8 h. The mixture was treated with 6 N HCl, until neutrality, and then was extracted thrice with EtOAc. The combined organic extracts were dried over Na_2_SO_4_ and the solution was concentrated in vacuum. The residue was purified on the silica column to give corresponding carboxylic acids.

DIBAL-H reduction. A solution of DIBAL-H (2.0 eq, 1.0 M in THF) was added dropwise to a solution of esters in dry THF (25 mL) at 0 C. The resulting mixture was stirred at room temperature. When the TLC shows the end of the substrate, the reaction was quenched by slow addition of a solution of saturated sodium potassium tartrate and, after dilution with CH_2_Cl_2_ stirred for 2 h. The mixture was partitioned thrice with CH_2_Cl_2_, and the combined organic extracts dried over Na_2_SO_4_.

#### 2.2.1 Synthesis of Biphenylethers 1-6

Esters 1 and 4 were synthesized according to the general procedure of Williamson, starting from 2-(chloromethyl)quinoline and methyl 4′-hydroxy-[1,1′-biphenyl]-3-carboxylate (13) or methyl 4′-hydroxy-[1,1′-biphenyl]-4-carboxylate (14).

Methyl 4'-(quinolin-2-ylmethoxy)-[1,1′-biphenyl]-3-carboxylate (1). Purification by flash column chromatography (silica gel, hexane: EtOAc 9:1 v/v) furnished compound 1 (quantitative yield). An analytic sample was further analyzed by HPLC on a Kinetex Biphenyl column (5 μm; 250 mm × 4.6 mm) in gradient (t_0_ = 20% ACN - t_5min_ = 20% ACN - t_20min_ = 95% ACN - t_25min_ = 95% ACN, flow rate 1 mL/min, t_R_ = 19.8 min). ^1^H NMR (400 MHz, CDCl_3_): δ 8.23 (1H, t, *J* = 8.0 Hz), 8.21 (1H, d, *J* = 8.4 Hz), 8.11 (1H, d, *J* = 8.6 Hz), 7.98 (1H, d, *J* = 8.0 Hz), 7.84 (1H, d, *J* = 7.9 Hz), 7.77 (1H, t, *J* = 8.6 Hz), 7.73 (1H, d, *J* = 8.4 Hz), 7.71 (1H, d, *J* = 8.0 Hz), 7.57 (1H, t, ovl), 7.57 (2H, d, *J* = 8.7 Hz), 7.48 (1H, t, *J* = 8.0 Hz), 7.13 (2H, d, *J* = 8.7 Hz), 5.45 (2H, s), 3.94 (3H, s). ^13^C NMR (100 MHz, CDCl_3_): δ 167.0, 158.2, 157.7, 147.5, 140.8, 137.1, 133.1, 131.0, 130.6, 129.8, 128.9, 128.8, 128.3 (2C), 127.8 (2C), 127.7, 127.6, 126.5, 119.1, 115.3 (2C), 71.4, 52.1. HRMS-ESI m/z 370.1441 [M + H^+^], C_24_H_20_NO_3_ requires 370.1438.

Methyl 4'-(quinolin-2-ylmethoxy)-[1,1′-biphenyl]-4-carboxylate (4). Purification by flash column chromatography (silica gel, hexane: EtOAc 9:1 v/v) furnished compound 4 (87% yield). An analytic sample was analyzed by HPLC on a Kinetex Biphenyl (5 μm; 250 mm × 4.6 mm) column in gradient (t_0_ = 20% ACN - t_5min_ = 20% ACN - t_20min_ = 95% ACN - t_25min_ = 95% ACN, flow rate 1 mL/min, t_R_ = 19.7 min). ^1^H NMR (400 MHz, CDCl_3_): δ 8.22 (1H, d, *J* = 8.6 Hz), 8.11 (1H, d, *J* = 8.2 Hz), 8.08 (2H, d, *J* = 8.6 Hz), 7.85 (1H, d, *J* = 8.2 Hz), 7.76 (1H, t, *J* = 8.2 Hz), 7.70 (1H, d, *J* = 8.6 Hz), 7.61 (2H, d, *J* = 8.6 Hz), 7.58 (2H, d, *J* = 8.9 Hz), 7.57 (1H, t, ovl), 7.13 (2H, d, *J* = 8.9 Hz), 5.46 (2H, s), 3.94 (3H, s). ^13^C NMR (100 MHz, CDCl_3_): δ 167.1, 158.6, 157.6, 147.5, 145.0, 137.1, 132.9, 130.1 (2C), 129.8, 128.9, 128.5 (2C), 128.3, 127.7, 127.6, 126.6 (2C), 126.5, 119.0, 115.3 (2C), 71.5, 52.1. HRMS-ESI m/z 370.1440 [M + H^+^], C_24_H_20_NO_3_ requires 370.1438.

4'-(quinolin-2-ylmethoxy)-[1,1′-biphenyl]-3-carboxylic acid (2) and (4'-(quinolin-2-ylmethoxy)-[1,1′-biphenyl]-3-yl)-methanol (3). Starting from ester 1, we performed NaOH hydrolysis and DIBAL-H reduction to obtain compounds 2 and 3, respectively.

4'-(quinolin-2-ylmethoxy)-[1,1′-biphenyl]-3-carboxylic acid (2). Purification by flash column chromatography (silica gel, DCM: MeOH 95:5 v/v) furnished compound 2 (quantitative yield). An analytic sample was analyzed by HPLC on a Kinetex Biphenyl (5 μm; 250 mm × 4.6 mm) column in gradient (t_0_ = 20% ACN - t_5min_ = 20% ACN - t_20min_ = 95% ACN - t_25min_ = 95% ACN, flow rate 1 mL/min, t_R_ = 17.0 min). ^1^H NMR (400 MHz, CDCl_3_): δ 8.29 (1H, t, *J* = 1.6 Hz), 8.23 (1H, d, *J* = 8.5 Hz), 8.14 (1H, d, *J* = 8.4 Hz), 8.03 (1H, d, *J* = 7.8 Hz), 7.85 (1H, d, *J* = 8.0 Hz), 7.79 (1H, d, *J* = 7.8 Hz), 7.77 (1H, t, *J* = 8.4 Hz), 7.72 (1H, d, *J* = 8.5 Hz), 7.58 (2H, d, *J* = 8.4 Hz), 7.57 (1H, t, ovl), 7.52 (1H, t, *J* = 7.8 Hz), 7.14 (2H, d, *J* = 8.4 Hz), 5.47 (2H, s). ^13^C NMR (100 MHz, DMSO-d6): δ 168.3, 159.0, 158.5, 147.9, 141.0, 138.2, 133.1, 132.4, 131.0, 130.3, 129.5, 129.0, 128.9, 128.6, 128.3, 127.8, 127.7, 127.6, 120.6, 116.5 (2C), 71.9. HRMS-ESI m/z 354.1137 [M-H^-^], C_23_H_16_NO_3_ requires 354.1136.

(4'-(quinolin-2-ylmethoxy)-[1,1′-biphenyl]-3-yl)-methanol (3). Purification by HPLC on a Nucleodur 100–5 (5 μm; 10 mm i. d. x 250 mm) with hexane/EtOAc 1:1 v/v as eluent (flow rate 3 mL/min, t_R_ = 20 min) gave compound 3 (quantitative yield). An analytic sample was analyzed by HPLC on a Kinetex Biphenyl (5 μm; 250 mm × 4.6 mm) column in gradient (t_0_ = 20% ACN - t_5min_ = 20% ACN - t_20min_ = 95% ACN - t_25min_ = 95% ACN, flow rate 1 mL/min, t_R_ = 17.2 min). ^1^H NMR (400 MHz, CDCl_3_): δ 8.22 (1H, d, *J* = 8.6 Hz), 8.12 (1H, d, *J* = 7.8 Hz), 7.85 (1H, d, *J* = 7.8 Hz), 7.76 (1H, t, *J* = 7.8 Hz), 7.71 (1H, d, *J* = 8.6 Hz), 7.57 (1H, t, *J* = 7.7 Hz), 7.55 (1H, s), 7.52 (2H, d, *J* = 8.6 Hz), 7.47 (1H, d, *J* = 7.6 Hz), 7.40 (1H, t, *J* = 7.6 Hz), 7.31 (1H, d, *J* = 7.6 Hz), 7.09 (2H, d, *J* = 8.6 Hz), 5.42 (2H, s), 4.76 (2H, s). ^13^C NMR (100 MHz, CDCl_3_): δ 158.1, 157.8, 147.5, 141.4, 140.9, 137.1, 133.9, 129.8, 128.9, 128.8, 128.3 (2C), 127.7, 127.6, 126.6, 126.0, 125.3 (2C), 119.1, 115.1 (2C), 71.2, 65.4. HRMS-ESI m/z 342.1487 [M + H^+^], C_23_H_20_NO_2_ requires 342.1489.

4'-(quinolin-2-ylmethoxy)-[1,1′-biphenyl]-4-carboxylic acid (5) and (4'-(quinolin-2-ylmethoxy)-[1,1′-biphenyl]-4-yl)-methanol (6). Starting from ester 4, we performed NaOH hydrolysis and DIBAL-H reduction in the same experimental conditions previously reported, to obtain compounds 5 and 6, respectively.

4'-(quinolin-2-ylmethoxy)-[1,1′-biphenyl]-4-carboxylic acid (5). Purification by flash column chromatography (silica gel, DCM: MeOH 95:5 v/v) furnished compound 5 (quantitative yield). An analytic sample was further analyzed by HPLC on a Kinetex Biphenyl (5 μm; 250 mm × 4.6 mm) column in gradient (t_0_ = 20% ACN - t_5min_ = 20% ACN - t_20min_ = 95% ACN - t_25min_ = 95% ACN, flow rate 1 mL/min, t_R_ = 16.9 min). ^1^H NMR (400 MHz, CD_3_OD+0.01% TFA): δ 9.23 (1H, d, *J* = 8.5 Hz), 8.42 (1H, d, *J* = 8.0 Hz), 8.39 (1H, d, *J* = 7.5 Hz), 8.24 (1H, t, *J* = 7.5 Hz), 8.23 (1H, d, *J* = 8.5 Hz), 8.10 (2H, d, *J* = 8.5 Hz), 8.03 (1H, t, *J* = 8.0 Hz), 7.76 (2H, d, *J* = 8.5 Hz), 7.73 (2H, d, *J* = 8.5 Hz), 7.32 (2H, d, *J* = 8.5 Hz), 5.80 (2H, s). ^13^C NMR (100 MHz, CDCl_3_): δ 168.7, 158.3, 157.3, 145.7, 145.0, 138.8, 133.2, 130.9, 130.4 (2C), 128.5 (2C), 128.4, 128.3, 127.8 (2C), 126.5 (3C), 119.2, 115.3 (2C), 69.8. HRMS-ESI m/z 354.1137 [M-H^-^], C_23_H_16_NO_3_ requires 354.1136.

(4'-(quinolin-2-ylmethoxy)-[1,1′-biphenyl]-4-yl)-methanol (6). Purification by flash column chromatography (silica gel, hexane: EtOAc 8:2 v/v) furnished compound 6 (92% yield). An analytic sample was analyzed by HPLC on a Kinetex Biphenyl (5 μm; 250 mm × 4.6 mm) column in gradient (t_0_ = 20% ACN - t_5min_ = 20% ACN - t_20min_ = 95% ACN - t_25min_ = 95% ACN, flow rate 1 mL/min, t_R_ = 19.4 min). ^1^H NMR (400 MHz, CDCl_3_): δ 8.22 (1H, d, *J* = 8.4 Hz), 8.11 (1H, d, *J* = 8.5 Hz), 7.85 (1H, d, *J* = 8.0 Hz), 7.76 (1H, t, *J* = 8.5 Hz), 7.71 (1H, d, *J* = 8.4 Hz), 7.57 (1H, t, *J* = 8.0 Hz), 7.55 (2H, d, *J* = 8.6 Hz), 7.53 (2H, d, *J* = 8.6 Hz), 7.42 (2H, d, *J* = 8.6 Hz), 7.10 (2H, d, *J* = 8.6 Hz), 5.44 (2H, s), 4.74 (2H, s). ^13^C NMR (100 MHz, CDCl_3_): δ 157.9, 157.8, 157.3, 147.5, 140.1, 137.1, 133.9, 129.8, 128.9, 128.5, 128.4, 127.7, 127.6, 127.5 (2C), 126.8 (2C), 126.5, 119.1, 115.2 (2C), 71.3, 65.1. HRMS-ESI m/z 342.1491 [M + H^+^], C_23_H_20_NO_2_ requires 342.1489.

#### 2.2.2 Synthesis of Ethers 7–12

Synthesis of compounds 17 and 18. To a solution of resorcinol in dry DMF methyl 5-bromopentanoate (0.5 eq) or methyl 4-bromobutanoate (0.5 eq) and K_2_CO_3_ (1 eq) were added and the reaction mixture was warmed to 100°C for about 12 h. After reagent consumption, the reaction mixture was cooled at RT, acidified with HCl 6N, and then DMF was evaporated under low pressure. The dry residue was extracted with ethyl acetate (3 × 50 mL). The organic phase was dried over Na_2_SO_4_, filtered and evaporated yielding a crude product that was then purified through flash silica column chromatography.

Methyl 5-(3-hydroxyphenoxy)-pentanoate (17). Purification by flash silica column chromatography in hexanes/EtOAc 8:2 afforded compound 17 (64%).

Methyl 4-(3-hydroxyphenoxy)-butanoate (18). Purification by flash silica column chromatography in hexanes/EtOAc 8:2 afforded compound 18 (43%).

Compounds 7 and 10 were synthesized, starting from 2-(chloromethyl)-quinoline by Williamson reaction, with analogous procedures to those detailed above for compounds 1 and 4.

Methyl 5-(3-quinolin-2-ylmethoxy)phenoxy)-pentanoate (7). Purification by flash silica column chromatography in hexanes/EtOAc 9:1 afforded compound 7 (75%). An analytic sample was further analyzed by HPLC on a Kinetex Biphenyl (5 μm; 250 mm × 4.6 mm) column in gradient (t_0_ = 20% ACN - t_5min_ = 20% ACN - t_20min_ = 95% ACN - t_25min_ = 95% ACN, flow rate 1 mL/min, t_R_ = 18.7 min). ^1^H NMR (400 MHz, CDCl_3_): δ 8.19 (1H, d, *J* = 8.5 Hz), 8.08 (1H, d, *J* = 8.5 Hz), 7.83 (1H, d, *J* = 8.2 Hz), 7.74 (1H, t, *J* = 8.5 Hz), 7.67 (1H, d, *J* = 8.5 Hz), 7.55 (1H, t, *J* = 8.1 Hz), 7.16 (1H, t, *J* = 8.2 Hz), 6.61–6.60 (2H, ovl), 6.51 (1H, dd, *J* = 8.2 Hz, 2.1 Hz), 5.37 (2H, s), 3.95 (2H, t, *J* = 7.0 Hz), 3.68 (3H, s), 2.39 (2H, t, *J* = 7.0 Hz), 1.80 (4H, pentet, *J* = 7.0 Hz). ^13^C NMR (100 MHz, CDCl_3_): δ 173.9, 160.3, 159.6, 158.0, 147.4, 137.2, 130.1, 129.9, 128.9, 127.8, 127.7, 126.6, 119.2, 107.6, 107.0, 101.9, 71.2, 67.3, 51.7, 33.7, 28.7, 21.7. HRMS-ESI m/z 365.1697 [M + H^+^], C_22_H_24_NO_4_ requires 365.1700.

Methyl 4-(3-(quinolin-2-ylmethoxy)phenoxy)-butanoate (10). Purification by flash silica column chromatography in hexanes/EtOAc 9:1 afforded compound 10 (72%). An analytic sample was analyzed by HPLC on a Kinetex Biphenyl (5 μm; 250 mm × 4.6 mm) column in gradient (t_0_ = 20% ACN - t_5min_ = 20% ACN - t_20min_ = 95% ACN - t_25min_ = 95% ACN, flow rate 1 mL/min, t_R_ = 18.2 min). ^1^H NMR (400 MHz, CDCl_3_): *δ* 8.20 (1H, d, *J* = 8.5 Hz), 8.10 (1H, d, *J* = 8.5 Hz), 7.83 (1H, d, *J* = 8.2 Hz), 7.74 (1H, t, *J* = 8.5 Hz), 7.68 (1H, d, *J* = 8.5 Hz), 7.56 (1H, t, *J* = 8.1 Hz), 7.16 (1H, t, *J* = 8.4 Hz), 6.62–6.60 (2H, ovl), 6.51 (1H, dd, *J* = 8.4 Hz, 2.3 Hz), 5.38 (2H, s), 3.98 (2H, t, *J* = 6.9 Hz), 3.68 (3H, s), 2.51 (2H, t, *J* = 6.9 Hz), 2.09 (2H, pentet, *J* = 6.9 Hz). ^13^C NMR (100 MHz, CDCl_3_): δ 173.7, 160.2, 159.6, 157.9, 147.4, 137.2, 130.1, 129.9, 128.9, 127.8, 127.6, 126.6, 119.2, 107.6, 107.1, 102.0, 71.2, 66.8, 51.7, 30.6, 24.7. HRMS-ESI m/z 352.1540 [M + H^+^], C_21_H_22_NO_4_ requires 352.1543.

NaOH hydrolysis of an aliquot of esters 7 and 10 in the same experimental condition furnished carboxylic acids (compounds 8 and 11, respectively).

5-(3-quinolin-2-ylmethoxy)phenoxy)-pentanoic acid (8). Purification by flash column chromatography (silica gel, DCM\MeOH 9:1 v/v) furnished compound 8 (68%). An analytic sample was further analyzed by HPLC on a Kinetex Biphenyl (5 μm; 250 mm × 4.6 mm) column in gradient (t_0_ = 20% ACN - t_5min_ = 20% ACN - t_20min_ = 95% ACN - t_25min_ = 95% ACN, flow rate 1 mL/min, t_R_ = 16.3 min). ^1^H NMR (400 MHz, CD_3_OD): δ 9.15 (1H, d, *J* = 8.5 Hz), 8.37 (1H, d, *J* = 8.5 Hz), 8.31 (1H, d, *J* = 8.2 Hz), 8.18 (1H, t, *J* = 8.5 Hz), 8.15 (1H, d, *J* = 8.5 Hz), 7.97 (1H, t, *J* = 8.1 Hz), 7.23 (1H, t, *J* = 8.2 Hz), 6.72–6.70 (2H, ovl), 6.63 (1H, dd, *J* = 8.2 Hz, 2.2 Hz), 5.68 (2H, s), 3.99 (2H, t, *J* = 6.5 Hz), 2.36 (2H, t, *J* = 6.5 Hz), 1.78 (4H, m). ^13^C NMR (100 MHz, CD_3_OD): δ 177.3, 161.8, 160.5, 158.4, 142.9, 133.4, 131.1 (2C), 129.6, 129.4, 129.3, 125.8, 121.1, 109.2, 108.0, 103.0, 70.1, 68.6, 34.5, 29.7, 22.7. HRMS-ESI m/z 350.1400 [M-H^-^], C_21_H_20_NO_4_ requires 350.1398.

4-(3-(quinolin-2-ylmethoxy)phenoxy)-butanoic acid (11). Purification by flash column chromatography (silica gel, DCM\MeOH 9:1 v/v) furnished compound 11 (97%). An analytic sample was further analyzed by HPLC on a Kinetex Biphenyl (5 μm; 250 mm × 4.6 mm) column in gradient (t_0_ = 20% ACN - t_5min_ = 20% ACN - t_20min_ = 95% ACN - t_25min_ = 95% ACN, flow rate 1 mL/min, t_R_ = 15.7 min). ^1^H NMR (400 MHz, CD_3_OD): δ 9.14 (1H, d, *J* = 8.5 Hz), 8.35 (1H, d, *J* = 8.5 Hz), 8.34 (1H, d, *J* = 8.2 Hz), 8.19 (1H, t, *J* = 8.5 Hz), 8.17 (1H, d, *J* = 8.5 Hz), 7.98 (1H, t, *J* = 8.1 Hz), 7.26 (1H, t, *J* = 8.5 Hz), 6.76–6.73 (2H, ovl), 6.66 (1H, dd, *J* = 8.5, 1.3 Hz), 5.68 (2H, s), 4.04 (2H, t, *J* = 6.4 Hz), 2.49 (2H, t, *J* = 6.4 Hz), 2.06 (2H, pentet, *J* = 6.4 Hz). ^13^C NMR (100 MHz, CD_3_OD): δ 175.4, 161.7, 160.3, 158.1, 144.8, 134.4, 131.1, 130.5, 130.3, 129.9, 129.3, 124.5, 120.8, 109.3, 108.1, 103.1, 69.2, 68.4, 31.3, 25.7. HRMS-ESI m/z 336.1245 [M-H^-^], C_20_H_18_NO_4_ requires 336.1241.

LiBH_4_ reduction. A solution of LiBH_4_ 2M in dry THF (2 eq) and dry MeOH (1 eq) was added to a solution of the esters 7 and 10 in dry THF at 0°C. The reaction was monitored *via* TLC and the substrate was fully converted after 5 h. The reaction was cooled to 0°C, quenched by adding a solution of NaOH 1N (2 eq), and stirred for 1 h. The mixture was then diluted with H_2_O and extracted with ethyl acetate (3 × 50 mL). The organic phase was dried over Na_2_SO_4_, filtered and evaporated yielding a crude product that was then purified through HPLC.

5-(3-(quinolin-2-ylmethoxy)phenoxy)-pentan-1-ol (9). Purification by flash silica column chromatography in hexanes/EtOAc 1:1 afforded compound 9 (80%). An analytic sample was analyzed by HPLC on a Kinetex Biphenyl (5 μm; 250 mm × 4.6 mm) column in gradient (t_0_ = 20% ACN - t_5min_ = 20% ACN - t_20min_ = 95% ACN - t_25min_ = 95% ACN, flow rate 1 mL/min, t_R_ = 16.5 min). ^1^H NMR (400 MHz, CDCl_3_): δ 8.20 (1H, d, *J* = 8.5 Hz), 8.11 (1H, d, *J* = 8.5 Hz), 7.84 (1H, d, *J* = 8.2 Hz), 7.75 (1H, t, *J* = 8.5 Hz), 7.68 (1H, d, *J* = 8.5 Hz), 7.56 (1H, t, *J* = 8.1 Hz), 7.16 (1H, t, *J* = 8.5 Hz), 6.61–6.60 (2H, ovl), 6.52 (1H, dd, *J* = 8.5 Hz, 2.4 Hz), 5.38 (2H, s), 3.94 (2H, t, *J* = 6.6 Hz), 3.67 (2H, t, *J* = 6.6 Hz), 1.80 (2H, pentet, *J* = 6.6 Hz), 1.63 (2H, m), 1.53 (2H, m). ^13^C NMR (100 MHz, CDCl_3_): δ 160.4, 159.7, 158.0, 147.5, 137.1, 130.1, 129.9, 129.0, 127.8, 127.7, 126.6, 119.2, 107.7, 107.0, 101.9, 71.3, 67.9, 62.9, 32.5, 29.0, 22.4. HRMS-ESI m/z 338.1753 [M + H^+^], C_21_H_24_NO_3_ requires 338.1751.

4-(3-(quinolin-2-ylmethoxy)phenoxy)-butan-1-ol (12). Purification by flash silica column chromatography in hexanes/EtOAc 4:6 afforded compound 12 (80%). An analytic sample was analyzed by HPLC on a Kinetex Biphenyl (5 μm; 250 mm × 4.6 mm) column in gradient (t_0_ = 20% ACN - t_5min_ = 20% ACN - t_20min_ = 95% ACN - t_25min_ = 95% ACN, flow rate 1 mL/min, t_R_ = 18.9 min). ^1^H NMR (400 MHz, CDCl_3_): δ 8.20 (1H, d, *J* = 8.5 Hz), 8.09 (1H, d, *J* = 8.5 Hz), 7.83 (1H, d, *J* = 8.2 Hz), 7.74 (1H, t, *J* = 8.5 Hz), 7.67 (1H, d, *J* = 8.5 Hz), 7.55 (1H, t, *J* = 8.1 Hz), 7.16 (1H, t, *J* = 8.3 Hz), 6.62–6.60 (2H, ovl), 6.52 (1H, dd, *J* = 8.3, 2.1 Hz), 5.38 (2H, s), 3.98 (2H, t, *J* = 6.0 Hz), 3.71 (2H, t, *J* = 6.3 Hz), 1.86 (2H, m), 1.74 (2H, m). ^13^C NMR (100 MHz, CDCl_3_): δ 160.2, 159.6, 157.9, 147.5, 137.1, 130.1, 129.8, 128.9, 127.8, 127.6, 126.6, 119.2, 107.7, 107.0, 101.9, 71.2, 67.8, 62.4, 29.5, 25.7. HRMS-ESI m/z 324.1590 [M + H^+^], C_20_H_22_NO_3_ requires 324.1594.

### 2.3 *In vitro* Assay

GPBAR1. To investigate the GPBAR1 activation, HEK-293 T cells were transiently transfected with Fugene HD reagent (Promega, Madison WI) using the following vectors: pCMVSPORT6-human GPBAR1, pGL4.29 (Promega, Madison WI), a reporter vector containing a cAMP response element (CRE) cloned upstream to the luciferase reporter gene luc2P and pGL4.70 (Promega, Madison WI), a vector encoding the human Renilla gene. At 24 h post transfection, the cells were stimulated with specific receptor agonist TLCA (10 μM) or compounds 1–12 at 10 μM. For the dose–response curve the cells transfected for GPBAR1 were stimulated with increasing concentrations (0.1–100 μM) of compound 2. At 18 h post stimulations, cellular lysates were assayed for luciferase and Renilla activities using the Dual-Luciferase Reporter assay system (E1980, Promega Madison WI). Luminescence was measured using Glomax 20/20 luminometer (Promega, Madison WI). Luciferase activities were normalized with Renilla activities.

Human CysLT_1_ (LTD4) (h) (antagonist effect) Cellular Functional Assay. These assays were performed at Eurofins Cerep-Panlabs (France). The cells were suspended in DMEM buffer (Invitrogen) and then distributed in microplates at a density of 3.104 cells/well. The fluorescent probe (Fluo4 Direct, Invitrogen) mixed with probenecid in Hank’s balanced salt solution (HBSS) buffer (Invitrogen) complemented with 20 mM Hepes (Invitrogen) (pH 7.4) was then added into each well and equilibrated with the cells for 60 min at 37°C then 15 min at 22°C. Thereafter, the assay plates are positioned in a microplate reader (CellLux, PerkinElmer), which was used for the addition of the test compound or HBSS buffer, then 5 min later, 0.1 nM LTD4 or HBSS buffer (basal control), and measured the changes in fluorescence intensity, which varies proportionally to the free cytosolic Ca^2+^-ion concentration. The results are expressed as a percent inhibition of the control response to 0.1 nM LTD4. The standard reference antagonist is MK 571. (Sarau et al., 1999; Fiorillo et al., 2021).

### 2.4 Computational Studies

CysLT_1_R. The crystal structure of the *Homo sapiens* Cysteinyl leukotriene receptor 1 (PDB ID 6rz4) (Luginina et al., 2019) was downloaded from the Protein Data Bank website. The soluble cytochrome b562 fragment and the co-crystallized ligand were removed and the residue Gln274 was reconstructed, whereas crystallographic water molecules within 5Å of the co-crystallized ligand were included. Residues protonation states were assigned in accordance with the most populated ones predicted by the H++ webserver (Anandakrishnan et al., 2012) at pH 7.4.

GPBAR1. The GPBAR1 homology model reported in D’Amore et al. (D’Amore et al., 2014) was employed for docking calculations. The receptor was prepared as in Biagioli et al. (Biagioli et al., 2020b).

Both receptors were treated with the Protein Preparation Wizard (Sastry et al., 2013) tool implemented in Maestro ver. 11.8.

Ligands. The 3D structure of 1–12 was built using the Graphical User Interface (GUI) of Maestro ver. 11.8 (Sepe et al., 2014). The protonation state of 1–12 at pH 7.4 in water has been calculated using the Epik module (Shelley et al., 2007). Finally, 1–12 were then minimized using the OPLS 2005 force field through 2500 iteration steps of the Polak-Ribiere Conjugate Gradient (PRCG) (Grippo and Lucidi 1997) algorithm.

Docking calculations of 1–12 were performed with the Glide software package, using the Standard Precision (SP) algorithm of the GlideScore function (Halgren et al., 2004) and the OPLS 2005 force field (Banks et al., 2005). A grid box of 25 × 16 × 17 Å for GPBAR1 receptor and one of 16 × 20 × 18 Å for CysLT_1_R centered on the ligand binding cavity were created. A total amount of 100 poses was generated and the conformational sampling of the ligand was enhanced by two times, as reported by the default setting of Glide. Docked conformations of 1–12 were then clustered based on their atomic RMSD. Globally, seven clusters were obtained and, among them, only the conformation included in the most populated cluster with both the Glide Emodel and GlideScore lowest-energy value was considered (Figure 3). All figures were rendered by UCSF Chimera (Pettersen et al., 2004).

### 2.5 Physiochemical Properties and Pharmacokinetic Characterization

Solubility. Ten microliters of a 10 mM solution of tested compounds were diluted either in 490 µL of PBS pH 7.4 or MeOH and agitated for 24 h at 250 rpm. The obtained solutions were first centrifuged for 5 min at 4,000 rpm and then further diluted adding 10 μL of each sample to 490 µL of MeOH. Samples were analyzed by LC-MS/MS and the ratio of mass signal area obtained in PBS and in organic solvent was then measured and used to determine solubility.

LogD. Measurements 40 µL of selected compounds were dissolved in 1960 µL of PBS pH 7.4/Octanol. Samples were shacked for 2 h at rt, 10 µL of each phase were withdrawn, diluted in 490 µL of MeOH, and analyzed by LC-MSMS. The ratio of mass signal area obtained in octanol and PBS at pH 7.4 and in organic solvent was employed to calculate LogD.

Metabolic Stability. All compounds were tested at the final concentration of 1 µM in a 50 mM potassium phosphate buffer (pH 7.4) containing 1% DMSO as vehicle. For microsomes assay, the incubation mixtures contained 0.15 mg of Human liver microsomes (Sigma-Aldrich, St. Louis, MO, United States), 5 mM MgCl_2_, 1 mM NADPH, 5 mM glucose 6-phosphate, 0.4 U mL^−1^ glucose 6-phosphate dehydrogenase. For S9 fraction analysis, the buffer contained 0.15 mg of S9 proteins (Sigma-Aldrich, St. Louis, MO, United States), 0.3 mM NADPH, 5.6 mM glucose-6-phosphate, 0.6 units/mL glucose-6-phosphate dehydrogenase, 5.8 mM UDP-glucuronic acid, 0.05 mM acetyl-CoA, 0.5 mM dithiothreitol, 0.5 mM 3′-phosphoadenosine 5′-phosphosulfate, 1 mM glutathione, 0.2 mM acetyl carnitine, 4 units/mL carnitine acetyl transferase, 0.5 mM glycine, and 0.5 mM taurine.

Samples were kept at 37°C and aliquots were removed at 0, 5, 15, 30, 45, 60, 90, 120, 150 min after protein additions. The reaction was stopped by adding 200 µL of ice-cold acetonitrile to withdrawn aliquots. After 2 h, samples were centrifuged for 10 min at 10,000 rpm, and supernatants were subjected to LC-MS/MS analysis.

The slope of the linear regression of the curve obtained reporting the natural logarithm of compound area versus incubation time (−k) was used in the conversion to *in vitro* t1/2 values by t1/2 = −ln (2)/k.

*In vitro* intrinsic clearance (Clint expressed as µL/min/mg) was calculated according to the following formula: Clint = Vx 0.693/t1/2 were V = volume of reaction (µL)/protein in the incubation (mg). Testosterone was used as a positive control for microsome and phase I enzymes, and 7-hydroxycoumarin was used as positive control for phase II enzymes.

### 2.6 *In vivo* Stability of Compound 2

The amount of compound 2 in plasma samples of animals treated with compound 2 was evaluated measuring the peak area of the compound at 1, 6, and 24 h after administration. 50 μL of plasma samples were mixed with 200 μL of acetonitrile, vortexed and incubated for 1 h on ice. Samples were then centrifuged for 10 min at 10,000 rpm and supernatants were evaporated to dryness. For LC/MS-MS analysis a QTRAP 6500 System (AB Sciex) equipped with Shimadzu LC-20A LC and AutoSampler was employed. Samples were dissolved in 100 μL of H_2_O, 30% MeOH, 0.2% FA, and the chromatographic separation was performed using a Luna Omega 3 µm Polar C18 100 × 2.1 mm (Phenomenex) and the best chromatographic results were achieved using the following gradient: 20% B from 0 to 1 min, 20–80% B from 1 to 5.0 min, 80–95% B from 5.0 to 5.1 min, held at 95% B for 3 min and then to 20% B (Buffer A: H_2_O 0.2% FA; Buffer B: MeOH 0.1% FA). The transition 356 →143 was employed for quantification. The following parameters were set: positive mode, DP 120 eV, EP 12 eV, CE 40 eV, CXP 23 V, CUR 30 psi, CAD Medium, IS 5500 V, TEM 350°C, GS1 and GS2 50 psi.

### 2.7 GEO Data Sets

The GSE135251 series includes gene expression profiles (RNA-seq analysis, Illumina NextSeq 500 system) of 216 snap frozen liver biopsies, comprising 206 NAFLD cases with different fibrosis stages and 10 controls (Govaere et al., 2020; Pfister et al., 2021).

### 2.8 Animal Model

C57BL/6J male mice were fed a high fat diet (HFD) containing 59 KJ% fat plus 1% cholesterol, w/o sugar (ssniff ^®^ EF R/M acc. D12330 mod. 22,7 ME/kg) and fructose (HFD-F) in drinking water (42 g/L), or normal chow diet for 61 days. Food intake was estimated as the difference of weight between the offered and the remnant amount of food at 3-days intervals. The food was provided as pressed pellets and the residual spillage was not considered. After 7 days, HFD-F mice were randomized to receive HFD-F alone or in combination with compound 2 (30 mg/kg/day) by gavage until the end of the experiment. Doses of each agent were chosen according to previously published data. Mice were housed under controlled temperature (22°C) and photoperiods (12:12-h light/dark cycle), allowed unrestricted access to standard mouse chow and tap water. The experimental protocol was approved by the Animal Care and Use Committee of the University of Perugia and by the Italian Minister of Health and Istituto Superiore di Sanità (Italy) and was in agreement with the European guidelines for use of experimental animals (permission n. 583/2017-PR). The general health of the animals was monitored daily by the Veterinarian in the animal facility. At the day of sacrifice, fed mice were deeply anesthetized with a mixture of tiletamine hypochloride and zolazepam hypochloride/xylazine at a dose of 50/5 mg/kg and sacrificed before 12 a.m.

### 2.9 Anthropometrical Determinations

Body weight and body length (nose-to-anus or nose–anus length) were measured in anesthetized mice at the time of sacrifice and were used to calculate the body mass index (BMI) (=body weight (g)/length^2^ (cm^2^)) and the Lee index (=cube root of body weight (g)/nose-to-anus length (cm)).

### 2.10 Biochemical Analyses

AST, ALT, total- and HDL-cholesterol, and triglycerides plasmatic levels were quantified using an automated clinical chemistry analyzer (Cobas, Roche).

### 2.11 Oral Glucose Tolerance Test and Insulin Levels

After 8 weeks of HFD-F mice were fasted overnight and orally administered glucose (1.5 g/kg body weight) for OGTT. The blood glucose concentrations were measured at 0, 15, 30, 60, 90, and 120 min after feeding or injection using a portable glucose meter (Accu-Check Go, Roche).

### 2.12 Thermal Images

Temperature of brown adipose tissue (BAT) was recorded through the study using a noninvasive technology. Briefly, mice were maintained at 25°C and the thermal images were taken by a FLIR E6 thermal imaging camera (FLIR System, Wilsonville, Oregon) and the surface temperature quantified by the FLIR Tools.

### 2.13 Histopathology

For histological examination, portions of the right and left liver lobes were fixed in 10% formalin, embedded in paraffin, sectioned and stained with Sirius red and/or Hematoxylin/Eosin (H&E), for morphometric analysis (Fiorucci et al., 2004). NASH severity (steatosis, hepatocytes ballooning, lobular inflammation, and portal inflammation) was scored in H&E-stained cross sections using an adapted grading system of human NASH as described previously (Carino et al., 2017b).

### 2.14 Quantitative Real-Time PCR Analysis

RNA was extracted from eWAT and colon with Direct-zol™ RNA MiniPrep w/Zymo-Spin™ IIC Columns (Zymo Research, Irvine, CA) and it was subjected to reverse transcription with FastGene Scriptase Basic Kit (Nippon Genetics Europe) in a 20 μL reaction volume.

For real time PCR, 50 ng cDNA were amplified in a 20 μL solution containing 200 nM of each primer and 10 μL of SYBR Select Master Mix (Thermo Fisher Scientific, Waltham, MA, United States). All reactions were performed in triplicate, and the thermal cycling conditions were as follows: 10 min at 95°C, followed by 40 cycles of 95°C for 10 s and 60°C for 45 s with QuantStudio™ 3 Real-Time PCR System (Applied Biosystems). The relative mRNA expression was calculated and expressed as 2−(ΔΔCt). Expression of the respective gene was normalized against Gapdh mRNA as an internal control. The following primers for Real-Time PCR were used: mouse-Gapdh: ctg​agt​atg​tcg​tgg​agt​cta​c and gtt​ggt​ggt​gca​gga​tgc​att​g; mouse-Srebf1: gat​caa​aga​gga​gcc​agt​gc and tag​atg​gtg​gct​gct​gag​tg; mouse-Fasn: tca​aga​tga​agg​tgg​cag​agg​tgc​t and ttg​agc​agt​gcc​ggg​att​cgg; mouse-Pgc1α: ctt​agc​act​cag​aac​cat​gca​g and aat​gct​ctt​cgc​ttt​att​gct​c; mouse-Fxr: act​gga​cca​cga​aga​tca​gat​t and gag​cgt​act​cct​cct​gag​tca​t; mouse-adiponectin: tga​cag​atc​agc​tcg​agt​gg and cag​tgc​cgt​cag​ttc​ttg​tg; mouse-Tnf-α: cca​cca​cgc​tct​tct​gtc​ta and agg​gtc​tgg​gcc​ata​gaa​ct; mouse-ucp2: ttg​ccc​gta​atg​cca​ttg​tc and gca​agg​gag​gtc​atc​tgt​ca; mouse-cd11b gtc​aga​gtc​tgc​ctc​cgt​gt and cag​ggt​cta​aag​cca​ggt​ca; mouse-pparγ: gcc​agt​ttc​gat​ccg​tag​aa and aat​cct​tgg​ccc​tct​gag​at; mouse-Il-1β: gct​gaa​agc​tct​cca​cct​ca and agg​cca​cag​gta​ttt​tgt​cg; mouse-Gcg: cca​aga​ttt​tgt​gca​gtg​gtt and cct​tca​gca​tgc​ctc​tca​aa.

### 2.15 AmpliSeq Transcriptome

High-quality RNA was extracted from mice livers, using the Direct-zol™ RNA MiniPrep w/Zymo-Spin™ IIC Columns (Zymo Research, Irvine, CA) according to the manufacturer’s instructions. RNA quality and quantity were assessed with the Qubit^®^ RNA HS Assay Kit and a Qubit 3.0 fluorometer (Invitrogen, Carlsbad, CA) followed by agarose gel electrophoresis. Libraries were generated using the Ion AmpliSeq™ Transcriptome Mouse Gene Expression Core Panel and Chef-Ready Kit (Comprehensive evaluation of AmpliSeq transcriptome, a whole transcriptome RNA sequencing methodology) (Thermo Fisher Scientific, Waltham, MA). Briefly, 10 ng of RNA was reverse transcribed with SuperScript™ Vilo™ cDNA Synthesis Kit (Thermo Fisher Scientific, Waltham, MA) before library preparation on the Ion Chef™ instrument (Thermo Fisher Scientific, Waltham, MA). The resulting cDNA was amplified to prepare barcoded libraries using the Ion Code™ PCR Plate, and the Ion AmpliSeq™ Transcriptome Mouse Gene Expression Core Panel (Thermo Fisher Scientific, Waltham, MA), Chef-Ready Kit, according to the manufacturer’s instructions. Barcoded libraries were combined to a final concentration of 100 p.m., and used to prepare Template-Positive Ion Sphere™ (Thermo Fisher Scientific, Waltham, MA) Particles to load on Ion 540™ Chips, using the Ion 540™ Kit-Chef (Thermo Fisher Scientific, Waltham, MA). Sequencing was performed on an Ion S5™ Sequencer with Torrent Suite™ Software v6 (Thermo Fisher Scientific, Waltham, MA). The analyses were performed with a range of fold <−2 and >+2 and a *p* value <0.05, using Transcriptome Analysis Console Software (version 4.0.1), certified for AmpliSeq analysis (Thermo-Fisher). The transcriptomic data have been deposited as dataset on Mendeley data repository (reference number linking to the repository: Fiorucci, Stefano; Biagioli, Michele; Marchianò, Silvia; Di Giorgio, Cristina (2021), “Discovery of potent dual GPBAR1/CysLT_1_R modulator for the treatment of metabolic fatty liver disease,” Mendeley Data, V1, doi: 10.17632/6dnrk9fc72.1).

### 2.16 Statistical Analysis

The ANOVA followed by nonparametric Mann-Whitney *U* test or a one-tailed unpaired Student t test were used for statistical comparisons (*p < 0.05) using the Prism 6.0 software (GraphPad).

## 3 Results

### 3.1 Synthesis

**FIGURE 2:**
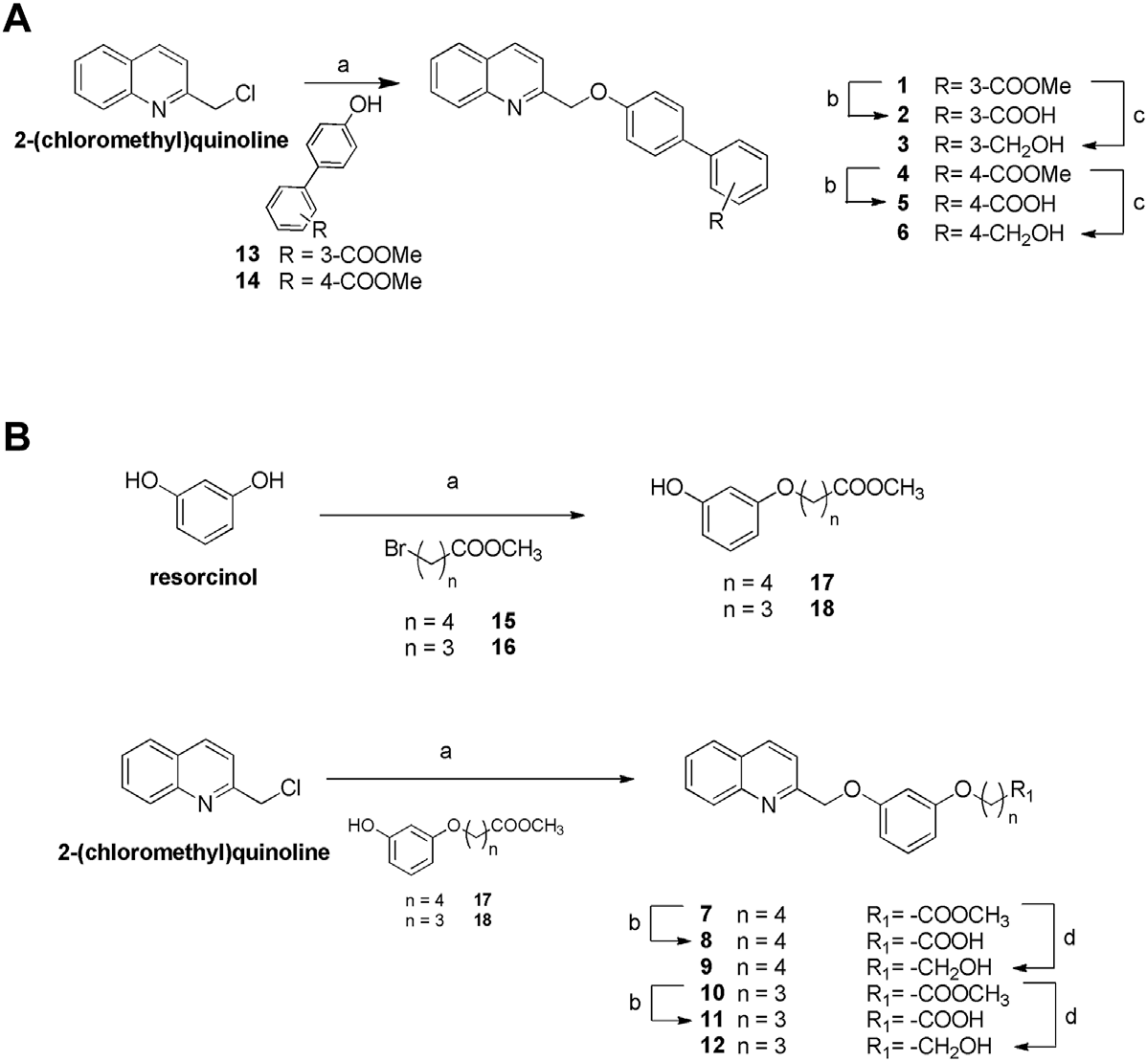
Synthesis of compounds 1–12. **(A)** Reagents and conditions. **a**) Compounds 13 or 14, K_2_CO_3_, dry DMF, 100°C, quantitative yield and 87%, respectively; **b**) NaOH in excess, MeOH: H_2_O 1:1 v/v, reflux, quantitative yield for both reactions; **c**) DIBAL-H, dry THF, 0°C, quantitative yield and 92%, respectively. **(B)** Reagents and conditions. **a**) K_2_CO_3_, dry DMF, 100°C; **b**) NaOH in excess, MeOH: H_2_O 1:1 v/v, reflux, 68 and 97%, respectively; **d**) LiBH_4_, dry CH_2_Cl_2_, 0°C, 80% for both reactions.

Figure 2 highlights the synthetic routes in obtaining derivatives 1–12, using few reaction steps and simple and cheap intermediates (i.e., chloromethylquinoline and resorcinol), with a view to future large-scale preparation, e.g., in a kilo lab, for future industrial applications.

Williamson reaction on the 2-chloromethylquinoline with methyl 4′-hydroxy- [1,1′-biphenyl]-3-carboxylate (13) or methyl 4′-hydroxy- [1,1′-biphenyl]-4-carboxylate (14) gave the methyl esters 1 (quantitative yield) and 4 (87% yield), respectively. Alkaline hydrolysis and DIBAL-H reduction furnished carboxylic acids 2 and 5 in quantitative yields and alcohols 3 and 6 (quantitative yield and 92%, respectively) (Figure 2A). For the preparation of compounds 7–12 (Figure 2B), first resorcinol was functionalized with the bromo alkyl methyl esters 15 and 16 by Williamson reaction to afford methyl esters 17 and 18 in fair yields (64 and 43%, respectively). Second, Williamson reaction between 17 or 18 with the commercially available 2-(chloromethyl)quinoline allowed the formation of the ether bridge in compounds 7 and 10 (75 and 72%, respectively). Basic hydrolysis and LiBH_4_ reduction gave the carboxylic acids (compounds 8 and 11, 68 and 97% respectively) or alcohols (compounds 9 and 12, 80% for both reactions).

### 3.2 *In vitro* Pharmacological Evaluation on the Whole Library

Derivatives 1–12 were tested for GPBAR1 activity, in a luciferase reporter assay with HEK-293T cells transfected with GPBAR1. Agonistic activities of compounds on GPBAR1 were compared to TLCA, which was set as 100%. All new compounds were screened *in vitro* for their CysLT_1_R antagonistic activity, as previously described (Sarau et al., 1999; Fiorillo et al., 2021). Compounds 2 and 7–12 were demonstrated antagonists of the CysLT_1_R, with efficacies in the range 76–109% and as shown in Table 1, the best results in terms of efficacy on both receptors were found in compound 2.

**TABLE 1:** *In vitro* evaluation of compounds 1–12.

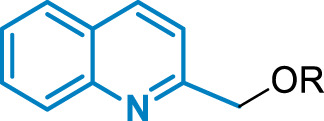

Compounds	GPBAR1^a^	EC_50_ ^b^ (μM)	CysLT_1_R^c^	IC_50_ ^b^ (μM)
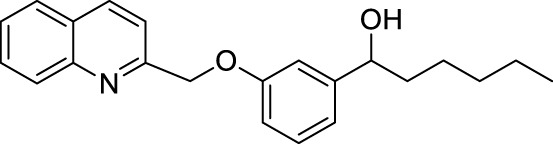	116.8 ± 0.2	1.1 ± 0.5	136.7 ± 27.8	2.5 ± 1.2
REV5901				
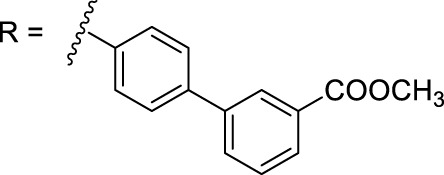	56.91 ± 7.8	-	21 ± 6.5	-
1				
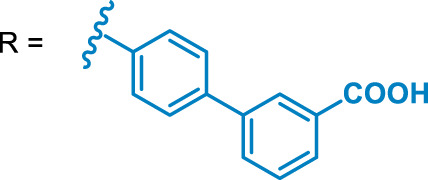	82 ± 1.2	4.6 ± 1.5	109 ± 1.6	5.67 ± 0.77
2				
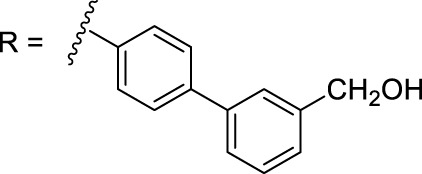	2.1 ± 0.06	-	59 ± 3.6	-
3				
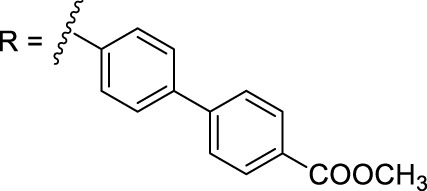	13.5 ± 1	-	13 ± 7.9	-
4				
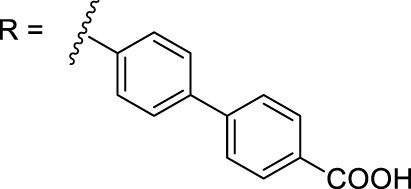	36.74 ± 3	-	43 ± 4.5	-
5				
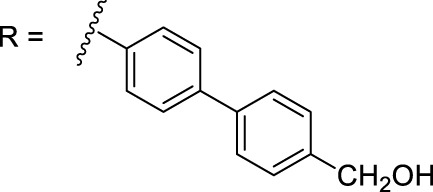	64.9 ± 13	-	0.8 ± 1.5	-
6				
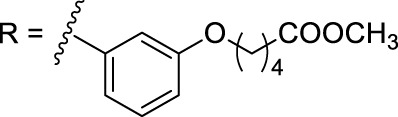	20.5 ± 2.4		76 ± 9.6	1.68 ± 0.28
7				
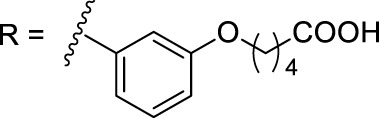	12 ± 1.3		99.6 ± 2.0	0.90 ± 0.50
8				
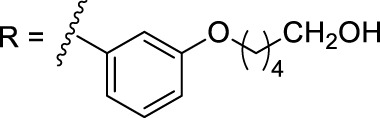	30.8 ± 7.5		106.9 ± 1.6	0.36 ± 0.58
9				
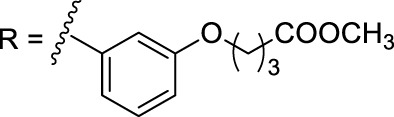	20.2 ± 4.5		92.1 ± 3.4	1.55 ± 0.43
10				
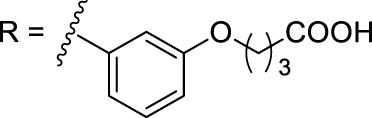	18.3 ± 4.8		92.8 ± 2.3	0.95 ± 0.2
11				
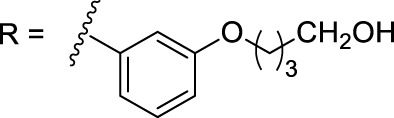	12.8 ± 3.3		108.6 ± 1.8	0.31 ± 0.24
12				

a Eff (%) is the maximum efficacy of the compound (10 µM) relative to TLCA (10 μM) as 100 in transactivation of a cAMP response element (CRE) on HEK293T cells; results are mean of at least two experiments ±SD.

b Results are mean of at least two experiments.

c These assays were performed by Eurofins Cerep-Panlabs (France).

### 3.3 Computational Studies

The binding modes of compounds 1–12 to GPBAR1 and CysLT_1_R were investigated by means of molecular docking calculations. This computational approach is commonly employed to elucidate the ligand/protein binding interaction and it has been successfully utilized by our group to characterize the binding mode of new compounds to several targets (Anzini et al., 2008; Nuti et al., 2010; Anzini et al., 2011; Troussicot et al., 2015; Limongelli 2020). In particular, we performed docking calculations using the Glide software package (see Methods for details) (Friesner et al., 2004; Halgren et al., 2004).

As regards CysLT_1_R, the crystallographic structure with PDB ID 6rz4 (Di Leva et al., 2019a) has been employed, while for GPBAR1, we used the 3D structure that has already been successfully employed in numerous drug design studies (D’Amore et al., 2014; Sepe et al., 2014; De Marino et al., 2017; Di Leva et al., 2019b) (see Methods for details).

Docking calculations of compounds 1–12 in GPBAR1 led to convergent results showing the quinoline group placed in the amphipathic pocket between transmembrane helices (TM) TM3 and TM5, making contacts with key residues known to participate in the GPBAR1 activation like Tyr89^3.29^, Asn93^3.33^, Phe96^3.36^, and Trp237^6.48^ (Macchiarulo et al., 2013; D’Amore et al., 2014; Di Leva et al., 2015; De Marino et al., 2017). On the other hand, the binding mode elucidated by docking calculations performed in CysLT_1_R showed the quinoline portion of compounds 1–12 positioned in the pocket formed by TM3, TM4, and TM5. In both cases, the ligand binding mode agrees with that we have recently reported for a series of alpha-pentyl-3-[2-quinolinylmethoxy] benzyl alcohol - REV5901–derivatives (Fiorillo et al., 2021). In the following section, we discuss in detail the binding mode of 2 that is the only dual-activity compound of the series and represents an interesting lead compound to achieve potent CysLT_1_R/GPBAR1 dual ligands.

#### 3.3.1 Docking Calculation in GPBAR1

**FIGURE 3:**
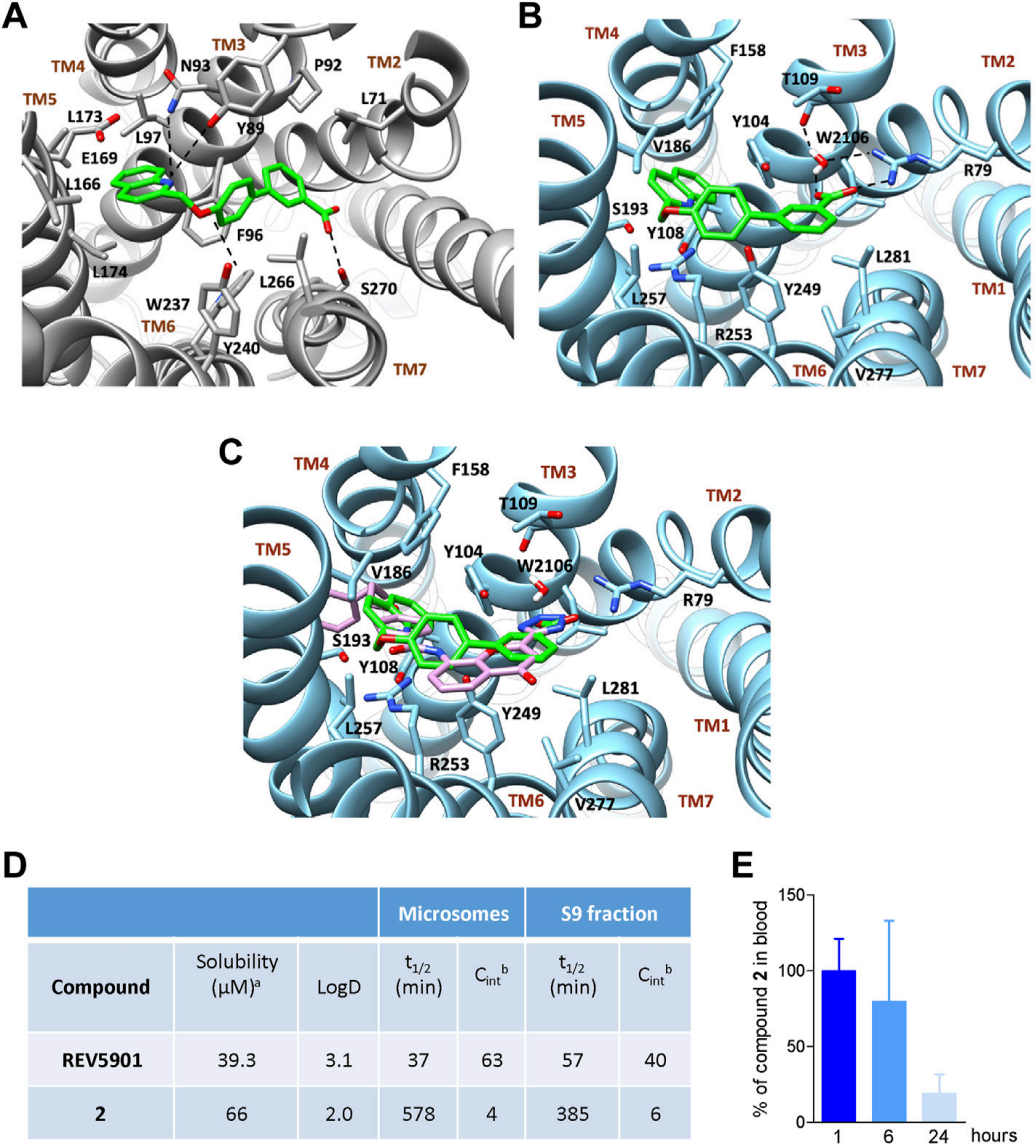
Binding mode in GPBAR1 and CysLT1R and in vitro and in vivo evaluation of the pharmacokinetics for compound 2. Binding mode of compound 2 (green stick) in GPBAR1 **(A)** (gray cartoon) and CysLT_1_R **(B)** (sky-blue cartoon). Superimposition between compound 2 (green stick) and the co-crystalized ligand pranlukast (plum stick) **(C)** in CysLT_1_R (sky-blue cartoon). The interacting residues of the receptor are shown in stick and labeled. TMs of the receptors are labeled. Oxygen atoms are depicted in red and nitrogens in blue. Hydrogens are omitted for the sake of clarity and H-bonds are displayed as black dashed lines. The bridging water molecule is reported as red sticks with explicit hydrogens. **(D)** *In vitro* pharmacokinetics for selected derivative. ^a^Aqueous solubility at pH 7.4; ^b^Reported as μl/min/mg protein. Results are mean of at least two experiments. **(E)** Measurement of the amount of compound 2 in the blood of mice at 1, 6, or 24 h after administration at the dose of 30 mg/kg. Results are the mean ± SD of 5 mice per group.

In the case of compound 2, the quinoline scaffold, located between TM3 and TM5, engages polar interaction with Tyr89^3.29^, Asn93^3.33^, and Glu169^5.38^. Furthermore, the quinoline moiety forms hydrophobic contacts with Phe96^3.36^, Leu97^3.37^, Leu166,^5.40^ Leu173^5.47^, and Leu174^5.48^ (Figure 3A). The ethereal oxygen H-bonds Tyr240^6.42^, while the biphenyl group extends through the binding site cavity shaped by Leu71^2.60^, Pro92^3.42^, Trp237^6.39^, Tyr240^6.42^, and Leu266^7.39^ allowing the terminal carboxylic group to form a H-bond with the side chain of Ser270^7.43^.

#### 3.3.2 Docking Calculation in CysLT_1_R

In CysLT_1_R, the quinoline group of compounds 1–12 occupies a region between TM3 and TM5, in a hydrophobic pocket made by Tyr108^3.37^, Phe158^4.60^, Val 186^5.35^, Ser 193^5.40^, and Arg253^6.55^ (Figure 3B). Here, Arg253^6.55^ and Phe158^4.60^ can form cation-π and π-π stacking interaction with the quinoline ring of compound 2, respectively, further stabilizing the binding mode. On the other hand, the biphenyl ring extends through the binding cavity, pointing toward TM2 and interacting with Tyr249^6.51^, Tyr104^3.33^, and Leu281^7.39^. Finally, the carboxy-terminal group forms a salt bridge interaction with Arg79^2.60^ and two water-mediated H-bonds, one again with Arg79^2.60^ and the other with Thr109^3.38^. Interestingly, the binding mode of compound 2 resembles the crystallographic binding pose of pranlukast, a potent antagonist of CysLT_1_R (Luginina et al., 2019), which occupies the same region of the receptor and interacts similarly with the surrounding residues of the pocket, including the H-bond and salt bridge interactions with Arg79^2.60^ (Figure 3C) (Fiorillo et al., 2021).

### 3.4 Pharmacokinetics Studies on Compound 2

Compound 2 was evaluated for physicochemical properties by LC-MS and compared with those of reference compound REV5901 (Fiorillo et al., 2021) (Figure 3D). Compound 2 showed a good aqueous solubility and a proper LOG_D_ at pH 7.4. To further assess the drug-likeness of selected compounds, the liability to hepatic metabolizing enzymes contained in microsomes and S9 fractions was evaluated *in vitro*, measuring by high-performance liquid chromatography-MS/MS the disappearance of unmodified compounds. Compound 2 revealed to be highly stable to Phase I enzymes contained in microsomal fraction. Therefore, employing liver S9 fraction, the metabolic stability of compound 2 was investigated also in the presence of enzymes responsible for Phase II reactions. Even in the presence of S9 contained enzymes derivative 2 was poorly modified and showed a t_1/2_ of 385 min (CL_int_ = 6). All these data highlight the pharmacological potential of compound 2, which associates the potency and the efficacy in dual modulation of GPBAR1/CysLT_1_R (Table 1) with excellent metabolic stability (Figure 3D).

### 3.5 *In vivo* Stability of Compound 2

Given the promising results obtained during *in vitro* pharmacokinetics studies, the profile of disappearance of compound 2 was also evaluated *in vivo*. Mice were treated with compound 2 and plasma samples withdrawn 1, 6, and 24 h after administration were analyzed by LC-MSMS to evaluate the concentration of compound 2. As shown in Figure 3E considerable percentage of compound 2 is still present 6 h after administration whereas approximately 20% is remaining after 24 h.

### 3.6 *In vivo* Pharmacological Evaluation on Compound 2, the Most Potent Dual GPBAR1/CysLT_1_R Modulator so far Identified

**FIGURE 4:**
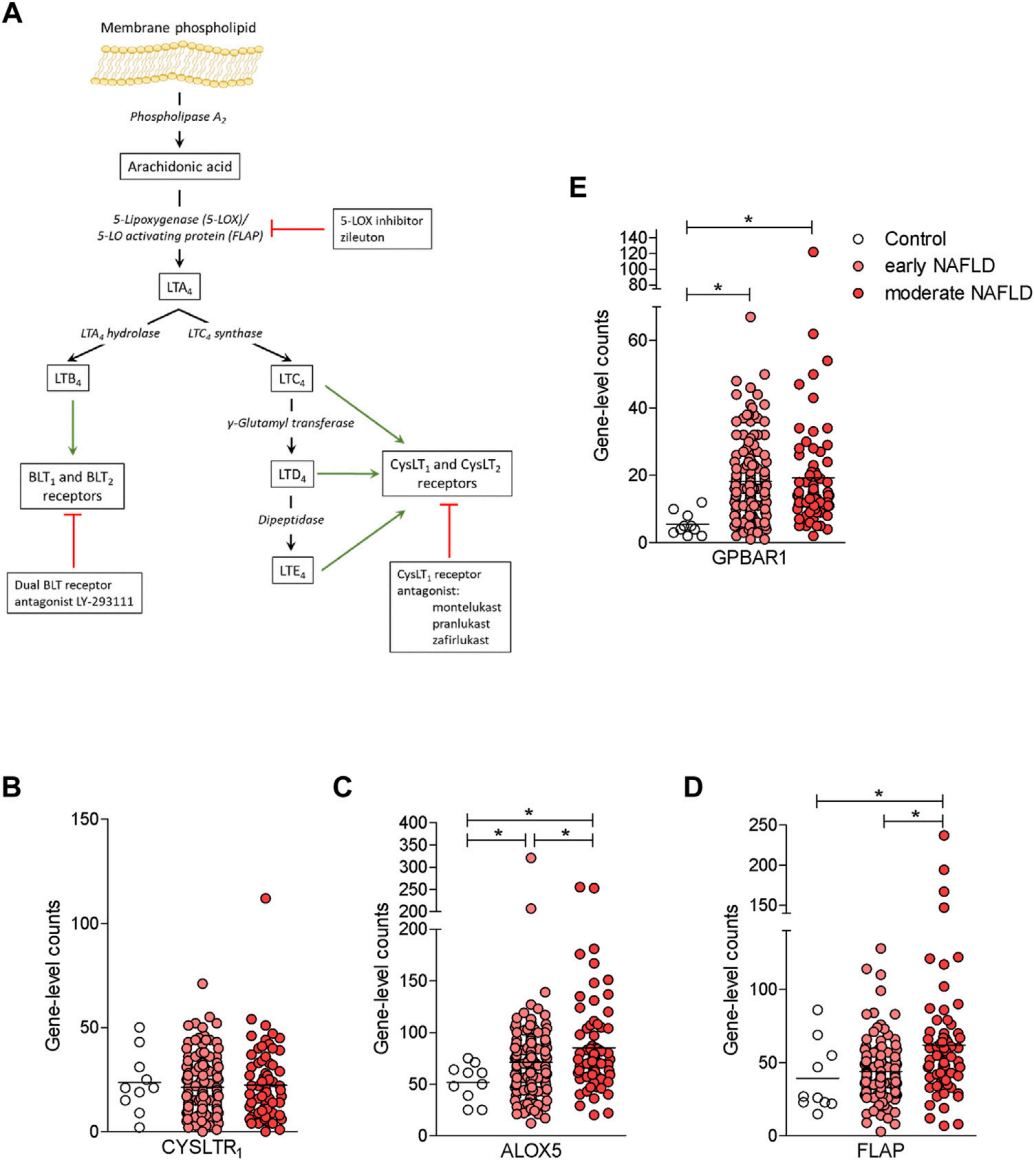
The synthesis of leukotrienes is upregulated in the liver of NAFLD patients. **(A)** Leukotriene synthesis pathway indicating the enzymes that are involved in each step of synthesis. **(B-E)** RNA-seq analysis of liver biopsy samples from GSE135251 repository of control, early NAFLD, and moderate NAFLD patients. Each dot represents a patient. Gene profile expression of **(B)** CYSLT_1_R, **(C)** ALOX5, **(D)** FLAP, and **(E)** GPBAR1. *p < 0.05.

Because compound 2 is a dual CysLT_1_R antagonist/GPBAR1 agonist and NAFLD/NASH is an orphan indication (Fiorucci et al., 2018), we have decided to investigate whether this novel chemical entity is effective in protecting against the development of steatosis and fibrosis in a rodent model of NASH. To validate the relevance of the CysLT_1_R/GPBAR1 in this model, we have first investigated whether the cystenyl-leukotriene pathway (Figure 4A) and that of GPBAR1 are modulated in the liver of NAFLD/NASH patients. To explore this concept, we accessed a GSE135251 repository (Govaere et al., 2020; Pfister et al., 2021) and assessed the expression of key genes in the two pathways in a large cohort of patients categorized as early and moderate NAFLD. As shown in Figures 4B–E, while the expression of CysLT_1_R mRNA was unchanged in the liver of these patients, the expression of GPBAR1 was robustly increased in both subsets of patients (Figures 4B,E). Furthermore, analysis of key genes in leukotrienes biosynthetic pathways (Martínez-Clemente et al., 2011) demonstrated that either the 5-lipoxygenase (ALOX5) or FLAP (also known as ALOX5AP) were robustly upregulated, and upregulation of FLAP followed the same trend of disease progression (Figures 4C,D).

**FIGURE 5:**
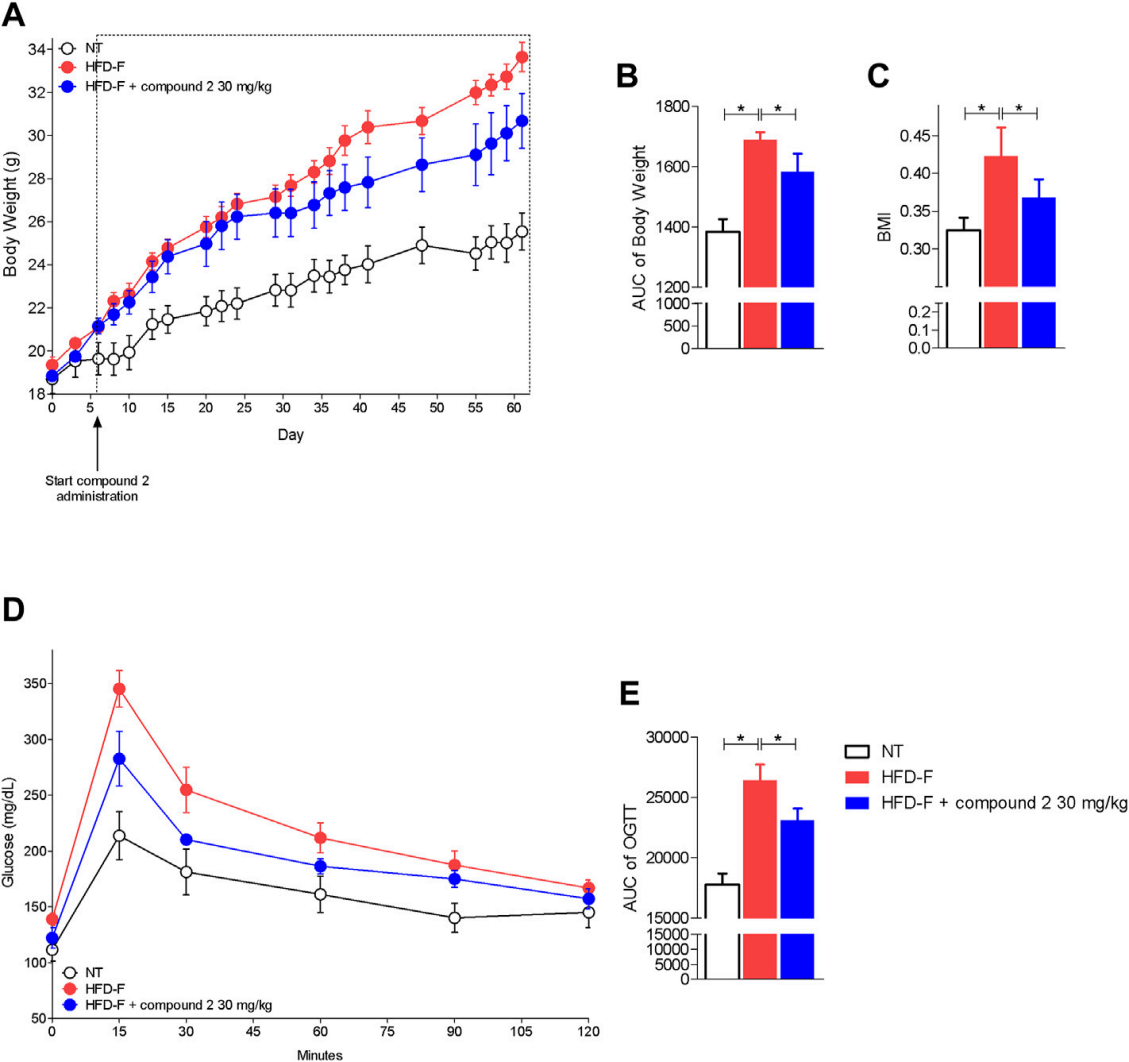
Beneficial effects of compound 2 on body weight gain and insulin sensitivity. C57BL/6 male mice were fed a high fat diet and fructose (HFD-F) or normal chow diet for 61 days. From day 7, compound 2 was administered by oral gavage at the dose of 30 mg/kg/daily. **(A)** Changes in body weight (%) and **(B)** AUCs of body weight expressed in arbitrary units. **(C)** Body mass index (BMI) at the end of the experiment. **(D)** Glucose plasma levels in response to OGTT and **(E)** AUCs of glucose plasma levels expressed in arbitrary units. Results are the mean ± SEM of 5-7 mice per group; *p < 0.05.

**FIGURE 6:**
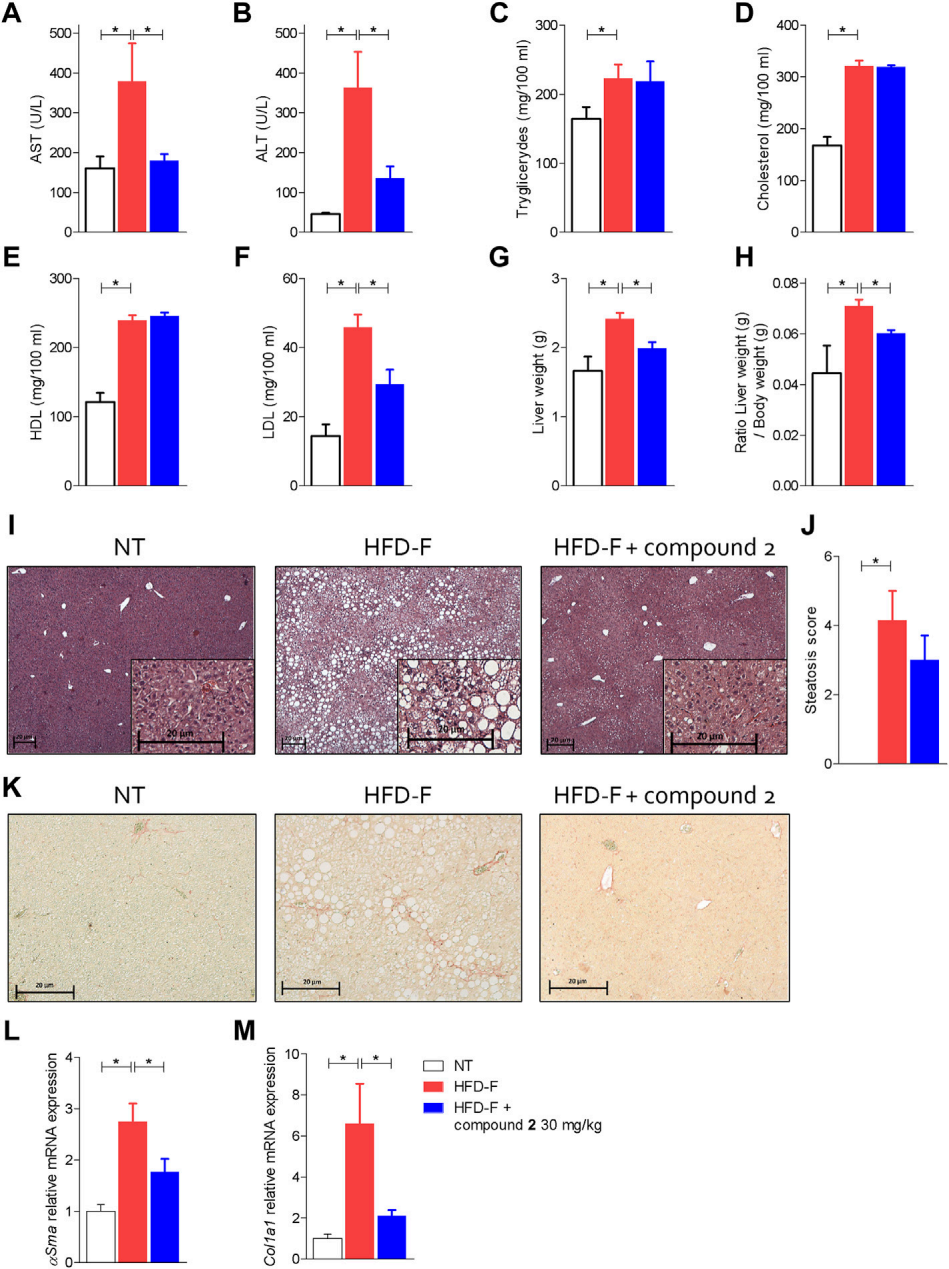
Beneficial effects of compound 2 on liver biochemistry and histopathology. C57BL/6 male mice were fed a high fat diet and fructose (HFD-F) or normal chow diet for 61 days. From day 7, compound 2 was administered by oral gavage at the dose of 30 mg/kg/daily. Plasmatic levels of **(A)** AST, **(B)** ALT, **(C)** Triglycerides, **(D)** Cholesterol, **(E)** HDL, and **(F)** LDL at the end of the study. Macroscopic and microscopic features of the liver: **(G)** liver weight (g), **(H)** ratio between liver weight (g) and body weight (g), **(I)** hematoxylin and eosin (H&E) staining of liver tissues obtained at the end of the study (magnification ×4, insets ×10) with **(J)** steatosis score, and **(K)** sirius red staining of liver tissues obtained at the end of the study (magnification ×10). Relative mRNA expression levels of **(L)** αSma and **(M)** Col1a1, measured in liver. Results are the mean ± SEM of 5-7 mice per group; *p < 0.05.

Since these human data demonstrated that either the leukotrienes pathway or GPBAR1 were upregulated in patients with NAFLD, we have decided to explore whether their modulation is beneficial in a model of NAFLD/NASH. We and others have previously reported that feeding mice a diet enriched in lipid and cholesterol and fructose in the drinking water (HFD-F) to simulate the high caloric intake caused by Western diet, leads to the development of liver injury showing the prototypical changes, steatosis, steatohepatitis, and fibrosis, detected at histopathology analysis in NASH patients (Carino et al., 2017a; Carino et al., 2019a; Carino et al., 2019b; Marchianò et al., 2022). In this experimental setting, HFD-F feed mice were treated orally with compound 2, 30 mg/kg for 54 days. As shown in Figure 5, feeding mice with HFD-F promoted the development of an obesogenic phenotype, with a 32% of weight gain in 61 days, which was partially reversed by compound 2. Importantly, treating mice with compound 2 not only reduced the body weight gain and body mass index (BMI) (Figures 5A–C), but also increased insulin sensitivity as demonstrated by the result obtained at the oral glucose tolerance test (OGTT) shown in Figures 5D,E), reduced AST and ALT plasma levels (Figures 6A,B), and partially reversed the proatherogenic lipid profile caused by feeding mice with an HFD-F (Figures 6C–F).

The analysis of the macroscopic characteristics of the liver (Figures 6G,H) at the end of the experiment showed an increase in liver weight in the animals treated with HFD-F compared to the control group. The administration of compound 2 reversed the effect exerted by the diet. The histopathology analysis of livers collected at the end of the study shown in Figures 6I,J demonstrated that while feeding mice an HFD-F promotes liver steatosis and hepatocytes ballooning (death), compound 2 was highly effective in reversing these features (Figures 6I,J). Additionally, Sirius red stained of liver sections revealed that feeding an HFD-F caused the development of a mild liver fibrosis and increased liver collagen deposition (Figure 6K), we found that these histopathology findings were robustly attenuated by feeding HFD-F mice with compound 2, which also reversed the liver fibrosis at molecular level. Thus, while HFD-F mice showed a robust increase in the liver expression of several pro-fibrogenic genes, αSma and Col1α1, this pattern was reversed by treating mice with compound 2 (Figures 6L,M).

**FIGURE 7:**
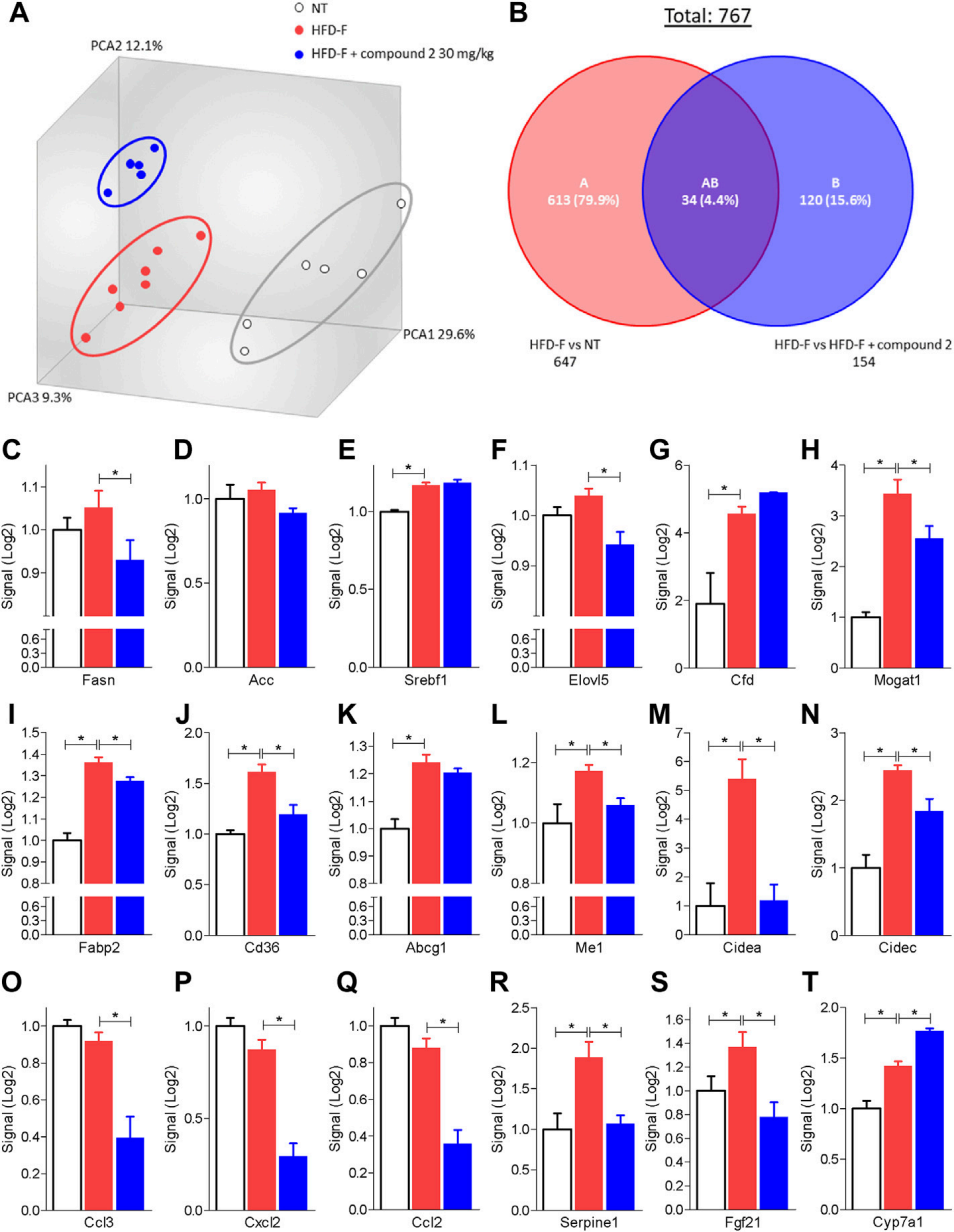
Liver transcriptome analysis of compound 2 in mice feed a HFD-F. C57BL/6 male mice were fed a high fat diet and fructose (HFD-F) or normal chow diet for 61 days. From day 7 compound 2 was administered by oral gavage at the dose of 30 mg/kg/daily. **(A)** Quantitative β analysis of PCoA that showed a major dissimilarity between the three experimental group and **(B)** Venn diagram of differentially expressed genes showing the overlapping regions between the experimental groups (fold change <−2 or >2, *p* value < 0.05). Relative mRNA expression levels extract from RNA-seq analysis of: **(C)** Fasn, **(D)** Acc, **(E)** Srebf1, **(F)** Elovl5, **(G)** Cfd, **(H)** Mogat, **(I)** Fabp2, **(J)** Cd36, **(K)** Abcg1, **(L)** Me1, **(M)** Cidea, **(N)** Cidec, **(O)** Ccl3, **(P)** Ccl2, **(Q)** Cxcl2, **(R)** Serpine1, **(S)** Fgf21 and **(T)** Cyp7a1. Results are the mean ± SEM of 5-7 mice per group; *p < 0.05.

**FIGURE 8:**
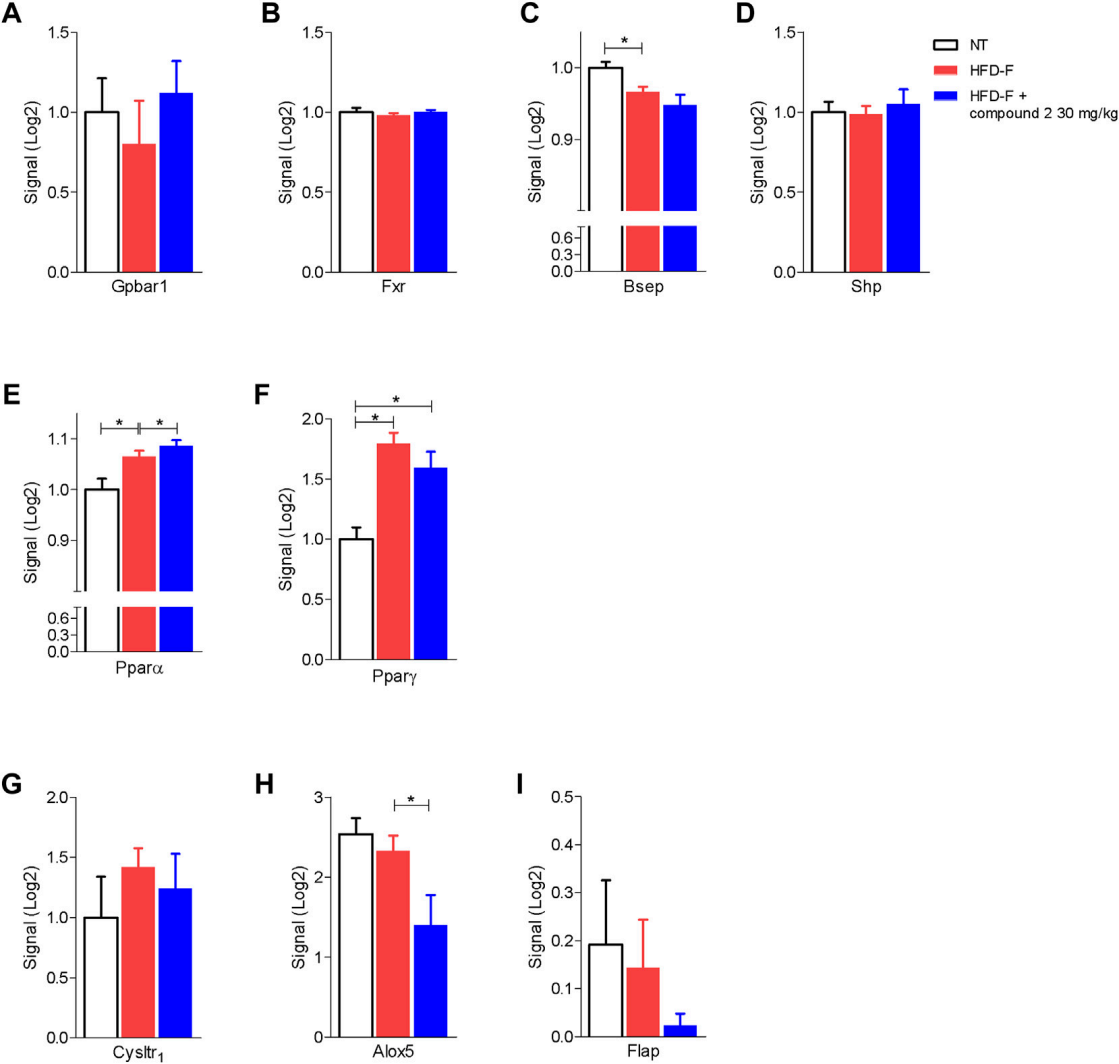
Effects of compound 2 on liver expression of GPBAR1, FXR, and its target genes and nuclear transcription factors. C57BL/6 male mice were fed a high fat diet and fructose (HFD-F) or normal chow diet for 61 days. From day 7 compound 2 was administered by oral gavage at the dose of 30 mg/kg/daily. Relative mRNA expression levels extract from RNA-seq analysis of: **(A)** Gpbar1, **(B)** Fxr, **(C)** Bsep, **(D)** Shp, **(E)** Pparα, **(F)** Pparγ, **(G)** CysLT_1_R, **(H)** Alox5, and **(I)** Flap. Results are the mean ± SEM of 5-7 mice per group; *p < 0.05.

The beneficial effects exerted by compound 2 on liver steatosis were manifested also at molecular levels, since treating HDF-F mice with the dual CysLT_1_R/GPBAR1 modulator reshaped the liver transcriptome, as shown in Figure 7. Specifically, treating mice with compound 2 resulted in robust remodeling of the expression of genes encoding for proteins involved in regulating cholesterol and triacylglycerol metabolism. The HFD-F diet compared to the untreated group modulated 647 genes of which 216 upregulated and 431 downregulated (Figure 7B and Supplementary Table S1). Among the genes most upregulated by HFD-F were many genes involved in the lipid metabolism such as Cfd (it is the most upregulated gene with a fold change of 105.43), Mogat1, Me1, Cidec, and Cidea (fold change of 5.67, 2.37, 23.38, and 7.63, respectively) and many genes of the Cyp family (Supplementary Table S1). The administration of compound 2 modulated 154 compared to the group subjected only to HFD-F (Figure 7B and Supplementary Table S2). As shown in Figures 7C–N, compound 2 exerted no effect on Srepb1f but reduced significantly Fasn, along with other genes, such as Elov5, Mogat1, fabp2, Cd36, Me1, and Cidea. Among the 154 genes modulated by compound 2 treatment compared to the group of mice subjected to HFD-F alone, the most downregulated gene is the chemokine Ccl3 with a fold change of–45.68 (Figure 7O and Supplementary Table S2). This finding is very interesting because elevated levels of Ccl3 have been documented in the serum and liver of patients with NASH and moreover the deletion of this gene protects mice from diet-induced steatohepatitis, hepatic fibrosis, and insulin resistance (Xu et al., 2021). Furthermore, among the genes most downregulated by compound 2 we also find the chemokines Cxcl2 and Ccl2 with a fold of–12.23 and–9.45, respectively (Figures 7P,Q) and Serpine 1 (Figure 7R). Importantly, compound 2 increased the expression of Cyp7a1, thus increasing bile acid synthesis and cholesterol disposal (Figure 7T), and decreased Fgf21, responsible for fatty acids and lipoproteins hepatic uptake (Figure 7S). Additionally, as shown in Figure 8, we found that compound 2 increased the expression of Pparα, a gene involved in fatty acid oxidation and Pparγ, in comparison to untreated mice, a validated target in NAFLD/NASH management. No differences are shown instead in the modulation of GPBAR1, FXR, and its target genes (Figures 8A–D). The cystenyl leukotriene pathway was also not modulated by HFD-F (Figures 8G–I). On the other hand, compound 2 statistically modulates only the expression of the Alox5 gene that was downregulated (Figure 8H).

### 3.7 Effects of Compound 2 on Adipose Tissues

**FIGURE 9:**
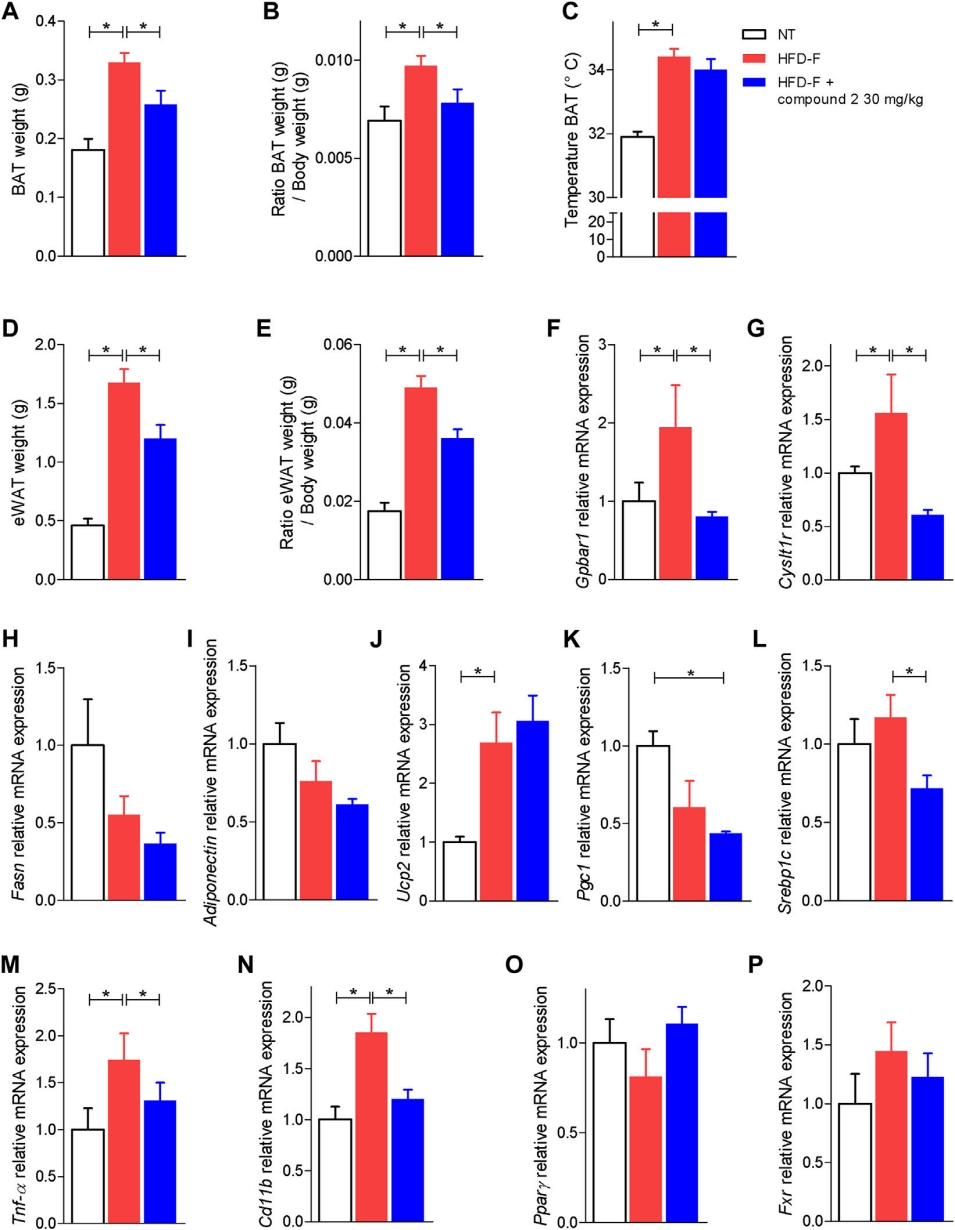
Effects of compound 2 on adipose tissue. C57BL/6 male mice were fed a high fat diet and fructose (HFD-F) or normal chow diet for 61 days. From day 7 compound 2 was administered by oral gavage at the dose of 30 mg/kg/daily. **(A)** BAT weight, **(B)** ratio BAT weight (g)/body weight (g), and **(C)** temperature of BAT (°C) on the day 51. **(D)** eWAT weight and **(E)** ratio eWAT weight (g)/body weight (g). Relative mRNA expression levels of **(F)** GPBAR1, **(G)** CysLT_1_R, **(H)** Fasn, **(I)** Adiponectin, **(J)** Ucp2, **(K)** Pgc1, **(L)** Srebp1c, **(M)** Tnf-α, **(N)** Cd11b, **(O)** Pparγ, and **(P)** Fxr in eWAT. Results are the mean ± SEM of 5-7 mice per group; *p < 0.05.

Because adipose tissues are a major therapeutic target in NASH, and both CysLT_1_R and GPBAR1 are expressed in this tissue, we have then assessed how compound 2 modulates the functionality of brown and white adipose tissue (BAT and WAT) (Scheja and Heeren 2016). The results shown in Figures 9A–C demonstrate that treating mice with compound 2 reduced the volume of BAT as well as the ratio of BAT weight/body weight. Because expansion of BAT is usually considered a protective event in the setting of high caloric intake, our results on BAT volume are likely due to reduction of body weight and BMI exerted by compound 2.

Because the WAT is the major storage tissue for fat, we have then examined whether treatment with compound 2 modulates WAT metabolism in the model. Exposure to the HFD-F significantly increased the ratio of epididymal white fat (eWAT) weight/body weight (Figures 9D,E) while the treatment with compound 2 reduced these changes. Feeding mice with the HFD-F increased the expression of GPBAR1 and CysLT_1_R (Figures 9F,G) and promoted inflammation as shown by increased levels of Tnf-α and Cd11b (Figures 9M–N). No statistically differences are shown in modulation of lipogenic genes (Figures 9H,I,L); feeding an HFD-F diet only shows a tendency to downregulate the expression of Pparγ and Pgc1α (Figures 9H-P). Treating mice with compound 2 reshaped the eWAT transcriptome and upregulated the expression of Ucp2, a marker of brown/beige trans-differentiation (Figure 9J).

Among the effects of GPBAR1 activation in the colon is the increase in GLP-1 secretion (Harach et al., 2012). Therefore, to confirm that the administration of compound 2 induced activation of GPBAR1, we measured the expression of the Gcg, gene encoding GLP-1, in the colon of mice. The data showed that compound 2 upregulated Gcg (Supplementary Figure S1B). The data also confirm the anti-inflammatory activity exerted by compound 2 also in the colon where it strongly reduced the expression of the pro-inflammatory cytokine Il-1β, whose expression was strongly increased by the HFD-F (Supplementary Figure S1A).

## 4 Discussion

In the present study, we report the synthesis and the pharmacological characterization of compound 2 as the first in a class of a novel series of orally active dual CysLT_1_R antagonists/GPBAR1 agonists showing efficacy in a validated model of NASH.

Compound 2 as all compounds described in this manuscript are easily accessible, using commercially available low-cost starting materials and few synthetic steps with excellent yields.

The structure–activity relationship (SAR) data in the present series of compounds was rationalized by in silico and pharmacological analyses. The dual activity toward two structurally different GPCRs was achieved building up on the scaffold of well characterized, thought abandoned, CysLT_1_R antagonist, the REV5901. In particular, a rigid spacer between the quinoline ring and the ending polar group (i.e., carboxylic or hydroxyl group), like the biphenyl moiety, is preferable over a flexible spacer (alkyl chain) to achieve a CysLT_1_R/GPBAR1 dual activity. In addition, the negatively charged carboxylic group allows establishing a strong salt bridge interaction with Arg79^2.60^ in the CysLT_1_R with respect to the hydroxymethyl and methoxycarbonyl groups. A similar interaction is formed by known CysLT_1_R antagonists, like pranlukast, which has a tetrazole group, “bioisoster” of the carboxylate group (Figure 3C). Such an interaction is weakened in case the carboxylic group is replaced by an alcohol or methoxycarbonyl group, as well as when the carboxylic group occupies a position in the ring different from the *meta*, like in the *para* substituted compound 5, where the interaction with Arg79^2.60^ of CysLT_1_R cannot be optimally oriented. Similarly, the binding mode of compound 2 in GPBAR1 explains its agonistic activity based on the contacts established with key residues known to participate in the GPBAR1 activation like Tyr89^3.29^, Asn93^3.33^, Phe96^3.36^, and Trp237^6.48^ (Di Leva et al., 2019a). In addition, the carboxylic group in *meta* position of 2 forms an H-bond with Ser270^7.43^, as seen in other GPBAR1 agonists (Sepe et al., 2014; Di Leva et al., 2015; De Marino et al., 2017; Di Leva et al., 2019b).

Because CysLT_1_R antagonists and GPBAR1 agonists have shown efficacy in treating NASH, we have further characterized the lead compound in this series for its efficacy toward a preclinical model of NAFLD/NASH. Compound 2 was administered orally for 9 weeks to mice fed on a high fat diet enriched in cholesterol. The results of these studies demonstrated that compound 2 effectively protected against body weight gain and the development of liver steatosis and hepatocytes injury as demonstrated by histopathology analysis and reduction of AST/ALT plasma levels. These beneficial effects were supported by a robust reshaping of the liver transcriptome. Thus, while exposure to the HFD-F modulated the expression of 767 genes in comparison to mice fed on a standard chow diet, this pattern was significantly remodeled by treating mice with compound **2,** which reshaped the expression of 154 genes. Further dissecting the transcriptome structure, we found that compound 2 effectively modulated the expression of key genes involved in the *de novo* lipogenesis including the Fasn, Fabp2, Mogat1 Cd36, Cidea and c and serpine1 and Pparα. PPARα is a validated target in the treatment of liver steatosis, and despite several single or dual PPARα/δ agonists have been proven effective in ameliorating insulin sensitivity and liver steatosis in preclinical models of NASH, clinical trials have shown that these agents hold limited efficacy in clinical settings, strongly supporting the notion that treatment of NASH requires a multitarget approach (Perakakis et al., 2021; Schattenberg et al., 2021; van den Hoek et al., 2021).

In addition to modulating lipogenetic pathways, compound 2 was highly effective in reducing the liver expression of biomarkers of inflammation including Ccl3, Ccl2, and Cxcl2. This family of chemokine has been shown to play a major role in developing inflammation and inflammation-driven fibrosis and CCL2 is a well-characterized target in the treatment of fibrosis in NASH patients. Cenicriviroc, the first in class of CCL2 receptor antagonist, has been found effective in reducing the progression of liver fibrosis (Ratziu et al., 2020). Of relevance, we have previously shown that GPBAR1 agonism in the rodent model of liver injury strongly inhibits CCL2/CCR5 expression, and that regulation of CCL2 is one of the mechanisms that support beneficial effects of GPBAR1 antagonism, suggesting that regulation of chemokine expression by compound 2 might be GPBAR1-dependent (Biagioli et al., 2017; Biagioli et al., 2020a).

We found that while compound 2 exerted no effect on FXR and its target genes Shp and Bsep, this agent potently increased the expression of Cyp7a1. The regulation of Cyp7a1 in this model of NAFLD (Fiorucci et al., 2021) is of relevance, since CYP7A1 is the rate-limiting enzyme in the synthesis of primary bile acids in the liver (Chiang and Ferrell 2020). We have recently shown that mice react to an HFD-F by increasing the rate of conversion of cholesterol into bile acids (Marchianò et al., 2022), and that this mechanism allows us to increase the fecal excretion of cholesterol, a protective mechanism that might contribute to limit liver fat accumulation in mice. Despite that regulation of CYP7A1 is different in human and mice (Fiorucci et al., 2021), there is robust evidence that CYP7A1 activation is protective against liver cholesterol accumulation (steatosis), inflammation, and fibrosis (Liu et al., 2016).

In addition to the liver, compound 2 exerted beneficial effects on adipose tissues. Indeed, while compound 2 reduces the BAT weight, likely because of its beneficial effects on body weight (Singh et al., 2021), it also promotes a series of changes on the eWAT (the mouse counterpart of the visceral fat in humans). Thus, not only compound 2 reduced eWAT weight but it also robustly reduced the expression of inflammatory genes such as Tnf-α and Cd11b, thus reversing the pro-inflammatory phenotype that is typically observed in NASH patients.

In summary, we have identified a novel family of dual CysLT_1_R antagonist and GPBAR1 agonist that exert beneficial effects in a mouse model of NASH. Genetic and pharmacological characterization of the lead compound in this series, compound 2, has shown that this novel approach allows the modulation of multiple targets in NASH, some of which are validated targets in the treatment of NASH. Together these data suggest that compound 2 is a promising drug candidate in treating NASH.

## Data Availability

The datasets presented in this study can be found in online repositories. The names of the repository/repositories and accession number(s) can be found in the article/Supplementary Material.

## Glossary

ALOX5,: arachidonate 5-lipoxygenase;
ALT,: alanine transaminase;
AST,: aspartate transaminase;
AUCs: areas under the curve;
BAT,: brown adipose tissues;
BMI,: body mass index;
CRE,: cAMP response element;
CysLT1R,: Cysteinyl Leukotriene Receptor 1;
CVD,: cardiovascular disease;
DIBAL-H,: diisobutylaluminum hydride;
DMSO,: dimethyl sulfoxide;
ESI-MS,: electrospray ionization mass spectrometry;
FLAP,: 5-lipoxygenase-activating protein;
GPCRs,: G-Protein Coupled Receptors;
GPBAR1,: G-Protein coupled Bile Acid Receptor 1;
HFD-F,: high fat diet and fructose;
HSC,: hepatic stellate cells;
LC-MSMS,: liquid chromatography-tandem mass spectrometry;
NAFLD,: nonalcoholic fatty liver disease;
NASH,: nonalcoholic steatohepatitis;
OGTT,: oral glucose tolerance test;
PGC-1α,: peroxisome-proliferator-activated receptor-γ coactivator-1 alpha;
PPARα,: peroxisome proliferator-activated receptor alpha;
SD,: standard deviation;
SEM,: standard error of mean;
T2DM: type 2 diabetes mellitus;
TLCA,: taurolithocholic acid;
Tnfα,: tumor necrosis factor alpha; \
UCP1,: uncoupling protein 1;
WAT,: white adipose tissues.
