# Supplementary figures and images for: Discovery of a Potent and Orally Active Dual GPBAR1/CysLT1R Modulator for the Treatment of Metabolic Fatty Liver Disease
Source: Front Pharmacol. 2022 Apr 25;13:858137. doi: 10.3389/fphar.2022.858137 (PMC9085577; doi:10.3389/fphar.2022.858137)

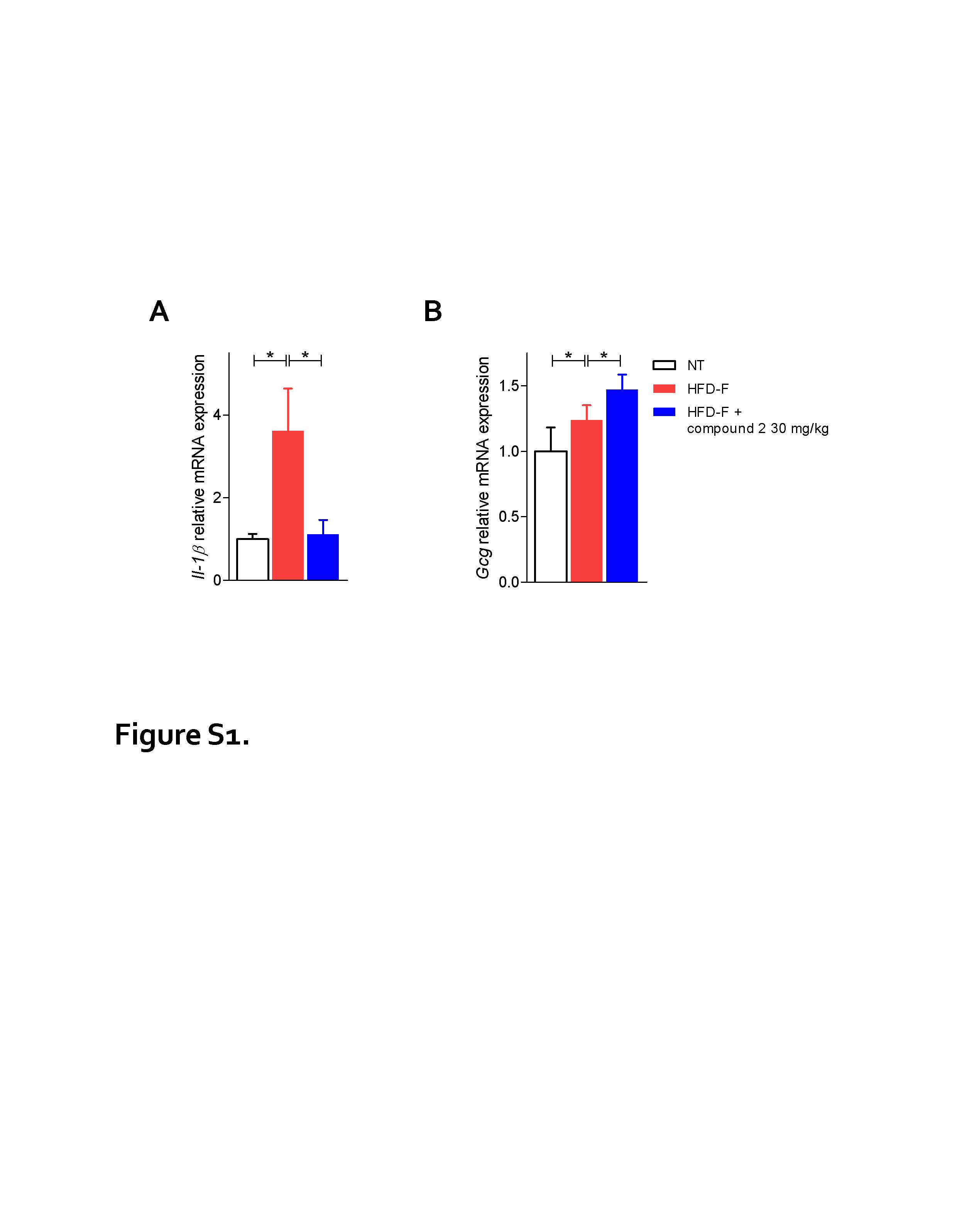

Supplement: Supplementary file 2 [file Image1.TIF]
